# eIF3f Mediates SGOC Pathway Reprogramming by Enhancing Deubiquitinating Activity in Colorectal Cancer

**DOI:** 10.1002/advs.202300759

**Published:** 2023-08-06

**Authors:** Qihao Pan, Fenghai Yu, Huilin Jin, Peng Zhang, Xiaoling Huang, Jingxuan Peng, Xiaoshan Xie, Xiangli Li, Ning Ma, Yue Wei, Weijie Wen, Jieping Zhang, Boyu Zhang, Hongyan Yu, Yuanxun Xiao, Ran‐yi Liu, Qingxin Liu, Xiangqi Meng, Mong‐Hong Lee

**Affiliations:** ^1^ Department of General Surgery The Sixth Affiliated Hospital Sun Yat‐sen University Guangzhou 510655 China; ^2^ Guangdong Provincial Key laboratory of Colorectal and Pelvic Floor Diseases The Sixth Affiliated Hospital Sun Yat‐sen University Guangzhou 510655 China; ^3^ Biomedical Innovation Center The Sixth Affiliated Hospital Sun Yat‐sen University Guangzhou 510655 China; ^4^ Department of Obstetrics and Gynecology The Sixth Affiliated Hospital Sun Yat‐sen University Guangzhou 510655 China; ^5^ Department of Clinical Biological Resource Bank Guangzhou Institute of Pediatrics Guangzhou Women and Children's Medical Center Guangzhou Medical University Guangzhou 510623 China; ^6^ Burn Plastic Surgery Yue bei People's Hospital Wujiang 512099 China; ^7^ State Key Laboratory of Oncology in South China & Collaborative Innovation Center of Cancer Medicine Sun Yat‐sen University Cancer Center Guangzhou 510060 China; ^8^ Department of Oncology The Sixth Affiliated Hospital Sun Yat‐sen University Guangzhou 510655 China

**Keywords:** colorectal cancer, eIF3f, FBXW7, MYC, PHGDH, ubiquitination, Wnt

## Abstract

Numerous studies have demonstrated that individual proteins can moonlight. Eukaryotic Initiation translation factor 3, f subunit (eIF3f) is involved in critical biological functions; however, its role independent of protein translation in regulating colorectal cancer (CRC) is not characterized. Here, it is demonstrated that eIF3f is upregulated in CRC tumor tissues and that both Wnt and EGF signaling pathways are participating in eIF3f's oncogenic impact on targeting phosphoglycerate dehydrogenase (PHGDH) during CRC development. Mechanistically, EGF blocks FBXW7β‐mediated PHGDH ubiquitination through GSK3β deactivation, and eIF3f antagonizes FBXW7β‐mediated PHGDH ubiquitination through its deubiquitinating activity. Additionally, Wnt signals transcriptionally activate the expression of eIF3f, which also exerts its deubiquitinating activity toward MYC, thereby increasing MYC‐mediated PHGDH transcription. Thereby, both impacts allow eIF3f to elevate the expression of PHGDH, enhancing Serine–Glycine–One–Carbon (SGOC) signaling pathway to facilitate CRC development. In summary, the study uncovers the intrinsic role and underlying molecular mechanism of eIF3f in SGOC signaling, providing novel insight into the strategies to target eIF3f‐PHGDH axis in CRC.

## Graphical abstract

Wnt pathways have activities in regulating eIF3f expression via β‐catenin and TCF4 signaling, and eIF3f is overexpressed in CRC. eIF3f acts as a deubiquitinating enzyme regulating PHGDH post‐transcriptionally through EGF‐GSK3β‐FBXW7β axis. Moreover, eIF3f can deubiquitinate and stabilize MYC, thereby regulating PHGDH transcriptionally via MYC. Therefore, targeting eIF3f‐PHGDH axis may be a potential CRC treatment strategy.

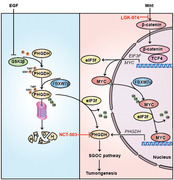

## 1 Introduction

Colorectal cancer (CRC) has a high mortality rate due to its strong resistance to therapies.^[^ ^1^ ^]^ Identifying the risk factors, including cancer biomarker,^[^ ^2^ ^]^ microbiome markers^[^ ^3^ ^]^ is increasingly urgent for CRC therapies. However, many uncharacterized cancer biomarkers remain to be identified in CRC for early detection and intervention.^[^ ^4^ ^]^ Defining the deregulations of these biomarkers can facilitate diagnosis and prognosis in CRC, and provide the potential therapeutic applications.

The eukaryotic translation initiation factor complex 3 (eIF3) which is a multiprotein complex known to be the largest initiation factors^[^ ^5^ ^]^ and may play a critical role in malignant transformation. Mammalian eIF3 is composed of 13 subunits (a‐m) and involves in almost all steps of translation initiation.^[^ ^6^ ^]^ Among the subunits of eIF3, eight subunits have PCI (proteasome, COP9 signalosome, eIF3) or MPN (Mpr1, Pad1, N‐terminal) domains and are recognized to form the structural core of eIF3.^[^ ^7^ ^]^ Notably, MPN domain‐containing proteins can be found in three protein complex involved in regulation of the protein synthesis and degradation, including the proteasome, COP9‐signalosome (CSN)^[^ ^8^ ^]^ and eukaryotic translation initiation factor 3 complexes, which determine the fates of cells. However, the functional roles of individual subunits of eIF3 have yet to be characterized, notably for its moonlighting roles independent of protein translation. Specifically, eIF3f, one of the subunits containing MPN domain of eIF3 complex, is essential for mice embryonic development as eIF3f‐knockout mice die at an early stage of development,^[^ ^9^ ^]^ suggesting its important biological functions. Accumulating evidences indicated that eIF3f expression is elevated in certain types of cancers, but decreased in other types of cancer.^[^ ^10^ ^]^ These findings suggest that the role of eIF3f involved in different cancers remained ambivalent and the mechanism associated with this deregulation is not fully understood.

Cancer metabolic reprogramming, one of the cancer hallmarks,^[^ ^11^ ^]^ involves tumor cells to rewire metabolic pathways to support rapid proliferation, continuous growth, metastasis, and resistance to therapies. Serine‐Glycine‐One‐Carbon (SGOC) pathway deregulation supports several metabolic processes that are crucial for the growth and survival of proliferating cells.^[^ ^12^ ^]^ Serine is a critical one‐carbon unit donor involved in methionine cycle and folate cycle, contributing to cancer growth,^[^ ^13^ ^]^ nucleotide synthesis,^[^ ^14^ ^]^ methylation reactions,^[^ ^15^ ^]^ and the generation of NADPH for antioxidant defense.^[^ ^16^ ^]^ Cancer cells particularly utilize serine as a major source of one‐carbon units for accelerating cell growth.^[^ ^17^ ^]^ PHGDH, the first enzyme of the SGOC pathway, involves in multiple cancers,^[^ ^17^, ^18^, ^19^ ^]^ but its mechanistic deregulation and clinical aggressiveness are not fully characterized. Therefore, understanding the importance of serine metabolism in cancer will provide new opportunities for therapeutic intervention.

The present study demonstrated that Wnt/β‐catenin/TCF4‐induced upregulation of eIF3f results in the SGOC pathway reprogramming and correlates with poor cancer survival in CRC, via regulating PHGDH and MYC stability through eIF3f's deubiquitinating activity, which in turn promotes cell growth and tumorigenicity. Thus, our data uncovers eIF3f is a critical cancer biomarker regulating SGOC pathway and provides novel insights into targeting eIF3f‐PHGDH axis as potential CRC treatment strategies.

## 2 Results

### 2.1 eIF3f is Overexpressed in Colorectal Cancer

**Figure 1:**
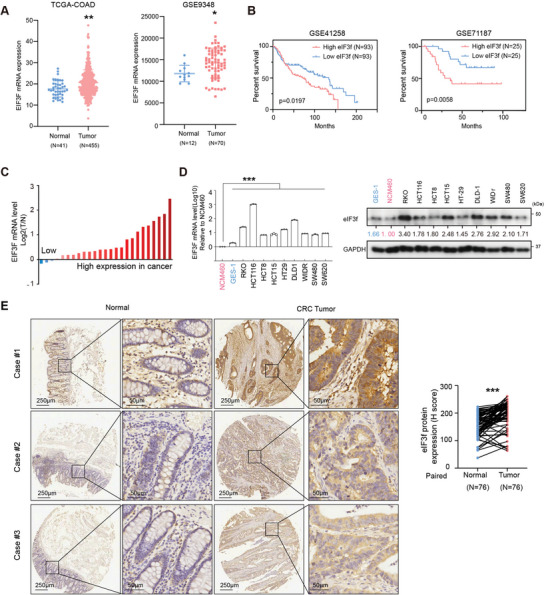
eIF3f is overexpressed in CRC. A) Expression level of eIF3f in colorectal tumor and normal tissues (TCGA‐COAD and GSE9348). Unpaired student's *t* test was performed. Solid lines denote the medians, the 5th and 95th percentiles. **p < 0.01, *p < 0.05. B) Kaplan‐Meier plot of overall survival time and log‐rank test based on EIF3F expression in GSE41258 and GSE71187 datasets. C) Waterfall plot of relative EIF3F mRNA levels of 26 paired samples of CRC and the adjacent normal tissue measured by qRT‐PCR. D) qRT‐PCR and immunoblotting of eIF3f expression in CRC and normal colonic cells (NCM460) and gastric epithelial cells (GES‐1). ***P<0.001. E) eIF3f is overexpressed in tumor than adjacent normal tissue. Analysis of eIF3f protein expression level in CRC and normal tissues by IHC staining of colorectal cancer tissue microarray (TMA). Representative different eIF3f staining images. The staining intensity and percentage were analyzed by Halo pathology software, paired *t*‐test were used, ***p < 0.001.

We have previously studied a MPN protein CSN subunit 6 and found that it is involved in cancer growth.^[^ ^8^, ^20^ ^]^ To further investigate another MPN domain‐containing protein, eIF3f, in CRC, we examined the status of eIF3f in the TCGA‐COAD database firstly, and found that eIF3f is overexpressed in CRC compared with normal tissues (**Figure** **1**A). Further data mining revealed that eIF3f is overexpressed in CRC compared with normal mucosa tissues in GSE9348 data sets (Figure 1A) and GSE77953 (Figure S1A, Supporting Information). Besides, Kaplan–Meier analyses of the data from CRC datasets GSE41258 and GSE71187 revealed that high eIF3f level correlated with poor survival (Figure 1B). Quantitative RT‐PCR (qRT‐PCR) analysis revealed that EIF3F mRNA level is high in CRC tissues compared to the adjacent normal mucosa tissues (Figure 1C). eIF3f was also highly expressed in all CRC cell lines (HCT‐116, RKO, HCT8, HCT15, DLD‐1, HT29, SW620, SW480, and WiDr cells) compared with the normal epithelial cells (NCM460 and GES‐1 cells) (Figure 1D). Additionally, an immunohistochemistry tissue microarray revealed that eIF3f expression in CRC was higher than that in normal tissue (Figure 1E). Therefore, eIF3f can be identified as a potential biomarker overexpressed in CRC patients.

To identify the role of eIF3f in CRC, we introduced shRNA‐mediated eIF3 knockdown (KD, Doxycycline‐induced) in HCT‐116 and RKO cells and validated the KD efficiency by westernblot and qRT‐PCR (Figure S1B,C, Supporting Information). Decreased expression of eIF3f inhibited CRC cell proliferation, colony formation (Figure S1D,E, Supporting Information) while overexpression of eIF3f promoted CRC cell colony formation (Figure S1F, Supporting Information), and eIF3f KD induced more apoptotic cells (Figure S1G, Supporting Information). These findings suggested an oncogenic role of eIF3f in controlling the CRC cell growth.

### 2.2 eIF3f Involves in Mitigating PHGDH, which is Overexpressed in CRC, Ubiquitination and Degradation

**Figure 2:**
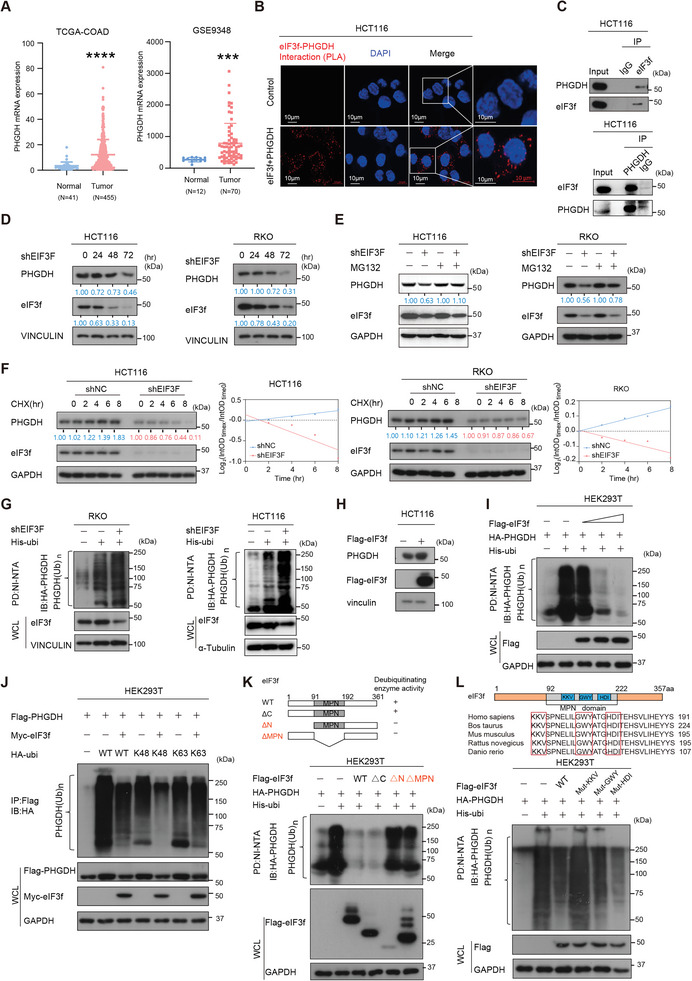
eIF3f deubiquitinates PHGDH. A) PHGDH mRNA expression was assessed from TCGA‐COAD and GSE9348 database. ***p < 0.001, ****p < 0.0001. B) Representative images of proximity ligation assay (PLA) results revealed that eIF3f interacted with PHGDH. The red signals demonstrate eIF3f‐PHGDH interaction. The nuclei of the cells were stained with DAPI (Blue signals). C) Endogenous co‐IP results indicated that EIF3F interacted with PHGDH. D) DOX‐inducible KD of eIF3f leads to downregulation of PHGDH. E) MG132 reversed EIF3F KD‐mediated PHGDH downregulation. F) eIF3F KD increased the turnover rate of PHGDH. G) Immunoblot analysis of poly‐ubiquitinated PHGDH in poly‐ubiquitination assays of indicated cells expressing DOX inducible shEIF3F and treated with 20 × 10^−6^ m MG132 for 6 h. The cell lysates were pulled down by nickel beads and immunoblotted with an anti‐ PHGDH antibody. H) eIF3F increased the steady expression of PHGDH. I) Overexpression of eIF3f reduced the ubiquitination level of PHGDH in a dose‐dependent manner. J) eIF3f deubiquitinated K48‐ubiquitin linkage of PHGDH. K) Schematic representation of vectors expressing WT or serial deletion mutants of Flag‐eIF3f (Upper panel). Deubiquitinating activity of different eIF3f mutants toward PHGDH ubiquitination. L) MPN mutations affects the deubiquitinating activity of eIF3f toward PHGDH ubiquitination. Schematic representation of the species alignment and the mutation sites (Upper panel). HEK293T cells co‐transfected with HA‐PHGDH and EIF3F wild‐type or mutants construct were treated with 50 × 10^−6^ m MG132 for 6 h before harvesting. Cells were lysed in guanidine‐HCl containing buffer and cell lysates were then pull down (PD) with nickel beads (NI‐NTA) and immunoblotted with HA‐PHGDH (lower panel).

Because eIF3f is highly expressed in CRC, we sought to investigate how eIF3f involved in regulating CRC progression. We immunoprecipitated Flag‐tagged eIF3f and identified eIF3f‐interacting partners using mass spectrometry. Among the top 20 interactors, most of the proteins are the subunits of eIF3 complex and proteasome complex, which indicate the success of our immunoprecipitation (Figure S2A, Supporting Information). Moreover, we had also conduced metabolomics analysis in HCT116 cells and the results showed that many metabolism pathways, including pyrimidine pathway, serine‐glycine pathway and pentose‐phosphate pathway, were significantly changed in the eIF3f KD group compared to the control group (Figure S2B, Supporting Information). These results suggested that eIF3f was associated with metabolism reprogramming in the progression of CRC. Among these pathways and interactome, Phosphoglycerate dehydrogenase (PHGDH) catalyzes the first step in serine synthesis pathway and was reported to regulate central carbon and nucleotide metabolism,^[^ ^14^ ^]^ while Carbamoyl‐phosphate synthetase 2, aspartate transcarbamylase, dihydroorotase (CAD) catalyzes the rate‐limiting step of pyrimidine synthesis.^[^ ^21^ ^]^ We further checked the expression of PHGDH and CAD in eIF3f KD CRC cells and the immunoblotting results showed that knockdown of eIF3f significantly reduced the expression of PHGDH, while exerted little effect on CAD (Figure S2C, Supporting Information). Importantly, PHGDH is highly expressed in CRC tumor tissues compared to normal tissues in TCGA‐COAD database and another two CRC datasets (TCGA‐COAD, GSE9348 and GSE41258) (**Figure** **2**A and Figure S2D Supporting Information). Further, we confirmed the direct binding of eIF3f‐PHGDH by proximity ligation assay (PLA) (Figure 2B), co‐immunoprecipitation (co‐IP) (Figure 2C), and immunofluorescence assay (Figure S2E, Supporting Information). The MPN domain (92‐222 aa) located in N‐terminal of eIF3f and functioned as deubiquitinating enzyme (DUB) activity.^[^ ^22^ ^]^ Based on the findings, it is possible that eIF3f may deubiquitinate PHGDH. Indeed, eIF3f KD leads to reduction of steady‐state expression of PHGDH (Figure 2D). Moreover, eIF3f KD‐mediated PHGDH downregulation could be rescued by the proteasome inhibitor MG132 (Figure 2E). In addition, eIF3f KD accelerates PHGDH protein turnover (Figure 2F), and leads to increased poly‐ubiquitination level of PHGDH (Figure 2G), while ectopic‐expression of eIF3f leads to increased steady‐state expression of PHGDH (Figure 2H) and reduced poly‐ubiquitination level of PHGDH in a dose‐dependent manner (Figure 2I).

Besides, eIF3f reduced ubiquitination of WT‐Ubi or K48 Ubi‐linked PHGDH but was unable to reduce K63 Ubi‐linked PHGDH (Figure 2J), indicating that eIF3f‐mediated deubiquitylation of PHGDH is a K48 linkage, which generally targets protein for degradation. eIF3f with MPN domain deleted (ΔMPN) or N‐terminal deleted (aa 1–91 deleted, ΔN) has attenuated its ability in decreasing PHGDH ubiquitination, suggesting that these domains are involved in DUB activity (Figure 2K). To further understand the mechanism that MPN domain is essential in deubiquitinating PHGDH, we constructed several MPN mutants of eIF3f by mutating residues conserved between CSN6 and eIF3f, including MPN‐KKV (160‐162), MPN‐GWY (170‐172) and MPN‐HDI (176‐178) (Figure 2L). These aa sequences were mutated to AAA. Immunoblotting showed that MPN‐KKV mutant (KKV→AAA) compromised its DUB activity in PHGDH ubiquitination; therefore, KKV residues are critical for deubiquitinating PHGDH (Figure 2L). Co‐IP studies showed that none of the mutants of eIF3f failed to bind PHGDH (Figure S2F, Supporting Information). These data showed that eIF3f attenuates ubiquitin‐mediated degradation of PHGDH through KKV residues in MPN domain, thereby increasing PHGDH protein stability.

To further investigate whether eIF3f regulated PHGDH expression also in a translation dependent way in CRC, we performed polysome profiling to determine the global mRNA translation activity in eIF3f KD and control cells. The results showed that there was no change of polysomes or monosome shift in eIF3f KD cells, compared to control cells and the qPCR results showed that there was no difference of PHGDH translation in eIF3f KD group, compared to the control group (Figure S2G, Supporting Information). We also measured the nascent protein synthesis with/without induction of eIF3f KD in HCT116 and RKO cells and the results showed that there was no significant difference of nascent protein synthesis in eIF3f KD group compared to the control group (Figure S2H, Supporting Information). Taken together, eIF3f deubiquitinates PHGDH independent of translation regulation.

### 2.3 FBXW7β‐Mediated PHGDH Ubiquitination through Binding the Phosphodegron of PHGDH in a GSK3β‐Dependent Manner

**Figure 3:**
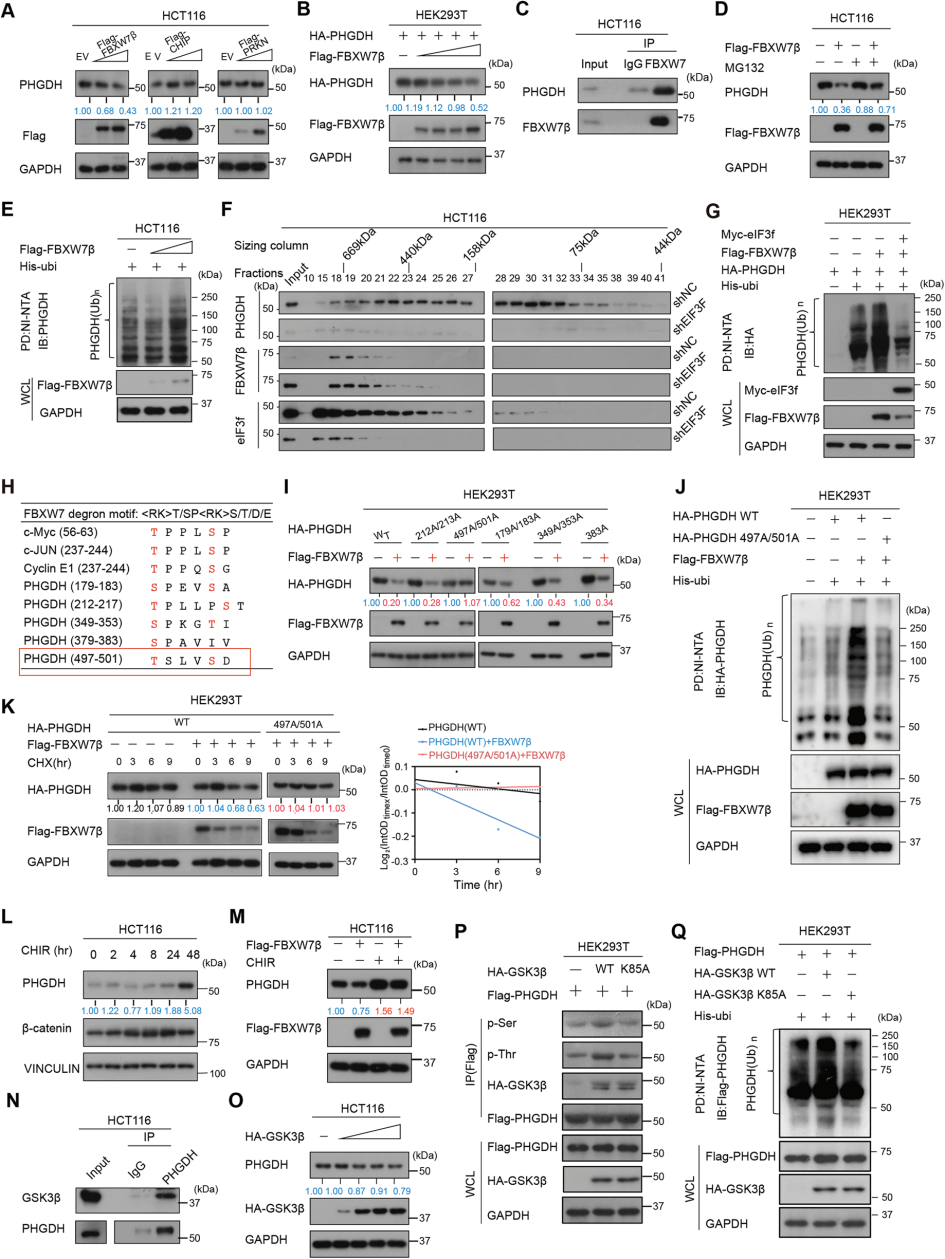
PHGDH is a substrate of FBXW7β. A) Screening of potential E3 ligases for PHGDH in HCT‐116 cells by transient transfection of indicated overexpression plasmids. B) FBXW7β decreases the steady state expression of PHGDH. C) Endogenous co‐IP result showed the interaction between FBXW7 β and PHGDH. D) MG132 reversed FBXW7β‐mediated PHGDH downregulation. E) FBXW7 β increases the poly‐ubiquitinated level of PHGDH. F) Gel‐filtration chromatography fraction analysis of cell lysates from shNC or shEIF3F HCT‐116 cells. Molecular size of eluted fraction is indicated above. G) eIF3f expression level affects FBXW7β‐mediated PHGDH polyubiquitination. H) Amino acid sequence of the putative FBXW7β binding motifs in PHGDH. I) 497A/501A mutant of PHGDH is not vulnerable to FBXW7β‐mediated downregulation. J) 497A/501A mutant of PHGDH is resistant to FBXW7β‐mediated ubiquitination. K) 497A/501A mutant of PHGDH is resistant to FBXW7β‐mediated acceleration of protein turn‐over. L) CHIR (GSK3β inhibitor) treatment increased PHGDH protein level. M) CHIR reversed FBXW7β ‐mediated PHGDH downregulation. N) Endogenous co‐IP results indicated that GSK3β interacted with PHGDH. O) GSK3β increases the steady‐state level of PHGDH. P) GSK3β kinase dead mutant (K85A) failed to enhance PHGDH phosphorylation. Q) GSK3β kinase dead mutant (K85A) failed to mediate PHGDH ubiquitination.

To further understand the mechanism of eIF3f‐mediated PHGDH deubiquitylation, we investigate the ubiquitin E3 ligase for PHGDH in CRC. To this end, we looked for the potential E3 ligases for PHGDH on the Ubi‐browser website (http://ubibrowser.bio‐it.cn/ubibrowser/) and chose the top five predicted E3 ligases of PHGDH for further investigation (Figure S3A, Supporting Information). Combined with the data mining (Figure S3B, Supporting Information) and immunoblotting results of the potential E3 ligases regulation on the steady state of PHGDH, we found that FBXW7β might be a potential E3 ligase of PHGDH (**Figure** **3**A and Figure S3C, Supporting Information). Indeed, FBXW7β ectopic‐expression decreased PHGDH steady‐state expression of protein (Figure 3B), but not mRNA (Figure S3D,E, Supporting Information). co‐IP studies indicated that PHGDH interacted with FBXW7β (Figure 3C and Figure S3F, Supporting Information). Further, FBXW7β‐mediated reduced expression of PHGDH could be rescued by MG132, suggesting that FBXW7β can regulate PHGDH through proteasome (Figure 3D). Indeed, FBXW7β increased ubiquitination of PHGDH (Figure 3E and Figure S3G, Supporting Information). Moreover, gel filtration studies indicate that eIF3f comigrates in complexes (about 440 Kd) with FBXW7β and PHGDH (Figure 3F), suggesting its role in regulating FBXW7β and PHGDH. We examined gel‐filtration chromatography fractions from sh‐NC and sh‐eIF3f expressing CRC cells and found that sheIF3f extracts had less PHGDH and more FBXW7β compared to the sh‐NC cell extracts side by side (Figure 3F). Further, co‐IP result confirmed the interaction between eIF3f and FBXW7β suggesting that eIF3f, FBXW7 and PHGDH may form a complex (Figure S3H, Supporting Information).

We next identify whether eIF3f could regulate FBXW7β‐mediated PHGDH ubiquitination. Immunoblotting revealed that overexpression of FBXW7β increased the ubiquitination level of PHGDH and this effect could be reversed in the presence of eIF3f (Figure 3G). Additionally, eIF3f KD increased the FBXW7β‐mediated ubiquitination of PHGDH (Figure S3I, Supporting Information). FBXW7β recognize its substrates through the presence of a conserved CDC4 phosphodegron (CPD) motif, which requires the substrate to be phosphorylated at specific residues in order to be ubiquitinated for further proteasome degradation (Figure 3H). Notably, protein sequence alignment analysis of known‐FBXW7 ubiquitinated proteins indicated that PHGDH has potential phosphorylation sites at Ser179/Ser183, Thr213/Ser217, Ser349/Thr353, Ser379, and Thr497/Ser501, which match the CPD motif (Figure 3H). Furthermore, NetPhos analysis demonstrated that Glycogen synthase kinase 3 beta (GSK3β) potentially phosphorylates PHGDH within these phosphodegron (Figure S3J, Supporting Information). Next we constructed the predicted potential functional mutant of PHGDH and found that PHGDH Mut(T497A/S501A) was resistant to downregulation by FBXW7β (Figure 3I), whereas WT PHGDH and other mutants were downregulated by FBXW7β. Moreover, FBXW7β increased the ubiquitination of WT PHGDH but failed to regulate PHGDH Mut(T497A/S501A) ubiquitination (Figure 3J). Furthermore, FBXW7β accelerated the turnover rate of the WT PHGDH but had little impact on PHGDH MUT(T497A/S501A) turnover (Figure 3K). These findings demonstrated that FBXW7β‐mediated downregulation of PHGDH requires the phosphorylation of PHGDH on phosphorylation degron motif (T497/S501), and eIF3f deubiquitinated FBXW7β‐mediated ubiquitination of PHGDH.

Next we further identify whether GSK3β is an important phosphorylation kinase of PHGDH. Since EGF can inactivate GSK3,^[^ ^23^ ^]^ it occurs that both EGF treatment (Figure S3K, Supporting Information) and GSK3 inhibitor (CHIR) treatment led to the increase of PHGDH (Figure 3L). Moreover, FBXW7β‐mediated PHGDFH downregulation was antagonized by CHIR treatment (Figure 3M). It is possible that FBXW7β‐mediated PHGDH degradation depends on GSK3β catalyzed‐phosphorylation. Co‐IP result identified that PHGDH interacted with GSK3β (Figure 3N), suggesting that PHGDH could be regulated by GSK3β. Indeed, PHGDH steady‐state expression decreased with increasing amount of GSK3β (Figure 3O). We further confirmed that the wild‐type GSK3β, but not the kinase dead‐mutant (GSK3β‐Κ85Α),^[^ ^24^ ^]^ significantly increased PHGDH phosphorylation (Figure 3P). Also, immunoblotting results showed that overexpression of GSK3β WT downregulated PHGDH in a dose‐dependent manner, while the GSK3β kinase dead mutant (K85A) had marginal effect on PHGDH (Figure S3L, Supporting Information). Moreover, we confirmed that only wild type GSK3β, but not the kinase dead‐mutant, could increase the ubiquitination of PHGDH (Figure 3Q). Taken together, these results indicated that GSK3β mediates PHGDH phosphorylation, which enhances the FBXW7β regulation on PHGDH ubiquitination.

### 2.4 eIF3f Antagonizes the Ubiquitination Level of MYC, a Transcriptional Activator of PHGDH, Thereby Promoting MYC‐Mediated Elevation of PHGDH

**Figure 4:**
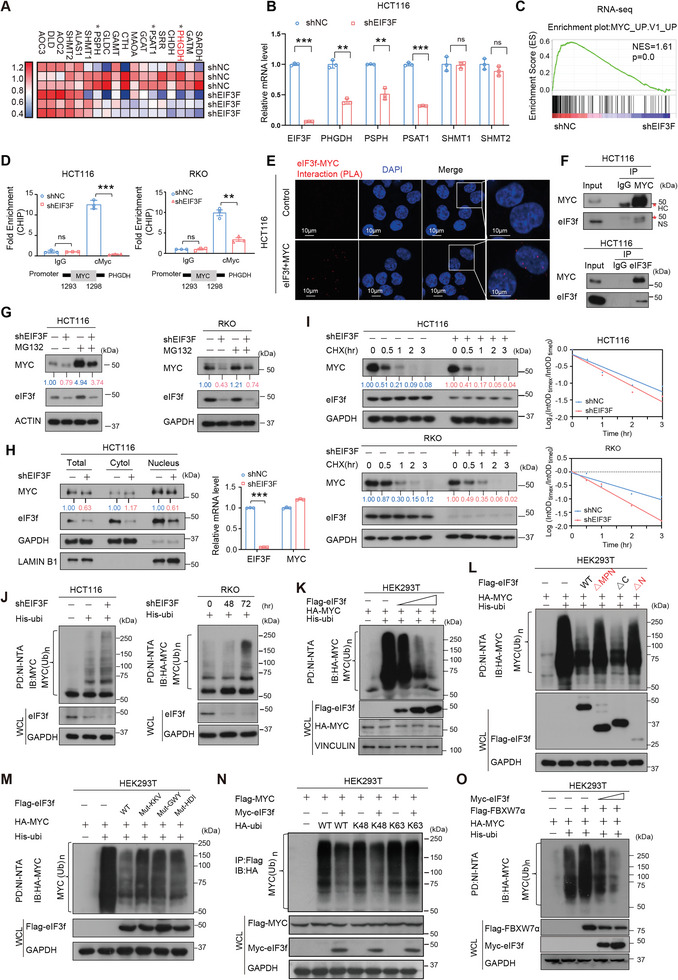
eIF3f regulated PHGDH transcription via stabilizing MYC. A) Heatmap of the expression of genes that mediate serine/one‐carbon metabolism in cells expressing shNC or shEIF3F in HCT‐116 cells. B) qRT‐PCR results showed the mRNA levels of genes related to serine synthesis pathway after eIF3f was knocked down. **P < 0.01, *** *P* <0.001, ns = no significant. C) GSEA analysis of RNA‐seq data revealed that eIF3f is associated with the expression of MYC‐targeted genes. D) ChIP‐PCR analysis revealed binding of transcription factor MYC on PHGDH promoter. MYC binds on the promoter region (1293bp‐1298 bp) of PHGDH. **P < 0.01, *** *P* <0.001. E) Representative images of proximity ligation assay (PLA) results revealed that eIF3f interacted with MYC. The red signals demonstrate eIF3f‐MYC interaction. The nuclei of the cells were stained with DAPI (Blue signals). F) HCT116 cell lysates were immunoprecipitated with either eIF3f or MYC antibody and immunoblotted with the indicated antibodies. IgG was used as a control. “* HC” indicated heavy chain. G) eIF3f KD leads to MYC downregulation. eIF3f was knocked down after DOX treatment. H) eIF3f KD leads to MYC downregulation in the nucleus. Cell lysates were harvested and separated into total lysates, cytoplasmic lysates and nucleus lysates via nucleus fraction followed by immunoblotting with indicated antibodies. mRNA expression of MYC is not changed after eIF3f was knocked down. ***P <0.001. I) Immunoblot analysis of the MYC protein turnover rate in indicated cells with eIF3f KD. Cycloheximide (CHX). eIF3f was knocked down after DOX treatment. J) Immunoblot analysis of poly‐ubiquitinated MYC in poly‐ubiquitination assays of indicated cells expressing DOX inducible shEIF3F and treated with 20 × 10^−6^ m MG132 for 6 h. The cell lysates were pulled down by nickel beads and immunoblotted with an anti‐MYC antibody. K‐L) HEK293T cells were transfected with increasing doses of Flag‐EIF3F, or EIF3F deletion constructs and His‐ubiquitin. The cell lysates were pulled down by nickel beads and immunoblotted with an anti‐MYC antibody. M) MPN mutations affects the deubiquitinating activity of eIF3f toward MYC ubiquitination. N) eIF3f deubiquitinates K48‐linked poly‐ubiquitination of MYC. O) eIF3f antagonizes FBXW7α‐mediated MYC ubiquitination.

To further investigate more detailed mechanisms that eIF3f involved in CRC progression, transcriptome analysis was performed after eIF3f was knocked down in HCT‐116 cells. Gene set enrichment analysis (GSEA) in RNA‐seq data demonstrated that many metabolic pathways, including glycine‐serine‐and‐threonine metabolism, were particularly enriched in eIF3f high expression group (**Figure** **4**A and Figure S4A, Supporting Information). qRT‐PCR analysis confirmed the downregulations of PHGDH, PSAT1, and PSPH from glycine‐serine‐and‐threonine metabolism in the eIF3f KD cells (Figure 4B and Figure S4B, Supporting Information). Furthermore, GSEA analysis also revealed that MYC target pathway related genes were downregulated in eIF3f KD group (Figure 4C). Interestingly, analyzing the TCGA‐COAD database (Figure S4C, Supporting Information) for the expression profiles of EIF3F/MYC and serine synthesis pathway related genes demonstrates a positive correlation between EIF3F/MYC expression and the expression of PHGDH, PSPH and PSAT1 (Figure S4D, Supporting Information). Given that MYC upregulates serine synthesis pathway related proteins, including PHGDH and PSAT1, based on *Eμ‐MYC* animal studies,^[^ ^25^ ^]^ we hypothesize that MYC may play a role in eIF3f KD‐mediated downregulation of PHGDH transcription expression.

To corroborate this idea, we performed Chromatin immunoprecipitation (ChIP) assay and the result showed that MYC could bind to the promoter region of PHGDH while knockdown of eIF3f could reduce the MYC binding to the PHGDH promoters (Figure 4D). Moreover, it turns out that eIF3f interacts with MYC directly based on proximity‐ligation assay (PLA), co‐IP assay and immunofluorescence in CRC cells (Figure 4E,F and Figure S4E, Supporting Information). Interestingly, immunoblotting showed that KD of eIF3f could reduce protein level of MYC but has no impact on MYC mRNA expression level (Figure 4G–H and Figure S4B, Supporting Information). Polysome profiling and qPCR results showed that there was no difference of Myc translation in eIF3f KD group, compared to the control group (Figure S2G, Supporting Information). eIF3f‐mediated MYC downregulation was rescued by MG132 (Figure 4G), suggesting that eIF3f may regulate proteasome‐mediated MYC ubiquitination/degradation. Furthermore, eIF3f KD accelerated the turnover rate of MYC and increased the ubiquitination level of MYC (Figure 4I,J), while overexpression of eIF3f reduced the ubiquitination level of MYC in a dose‐dependent manner (Figure 4K). In line with previous findings, neither eIF3f‐ΔMPN nor eIF3f‐ΔN remained its ability in decreasing MYC ubiquitination (Figure 4L), suggesting that these domains also involved in DUB activity toward MYC. Moreover, MPN‐KKV mutant of eIF3f also compromised its DUB activity in MYC ubiquitination; therefore, KKV residues are critical for deubiquitinating MYC (Figure 4M). Co‐IP experiment results showed that none of the mutants of eIF3f failed to bind MYC (Figure S4F, Supporting Information). Importantly, eIF3f also deubiquitinated K48‐linked ubiquitination of MYC but not K63‐linked ubiquitination of MYC (Figure 4N). Further, we demonstrated that eIF3f antagonized the FBXW7α‐mediated ubiquitination level of MYC (Figure 4O). All these data demonstrated that eIF3f‐regulated PHGDH mRNA expression can be executed via antagonizing the ubiquitination level of MYC.

### 2.5 Knockdown of eIF3f Inhibited Serine Synthesis Pathway in CRC Cells and Suppressed Tumor Growth In Vivo

**Figure 5:**
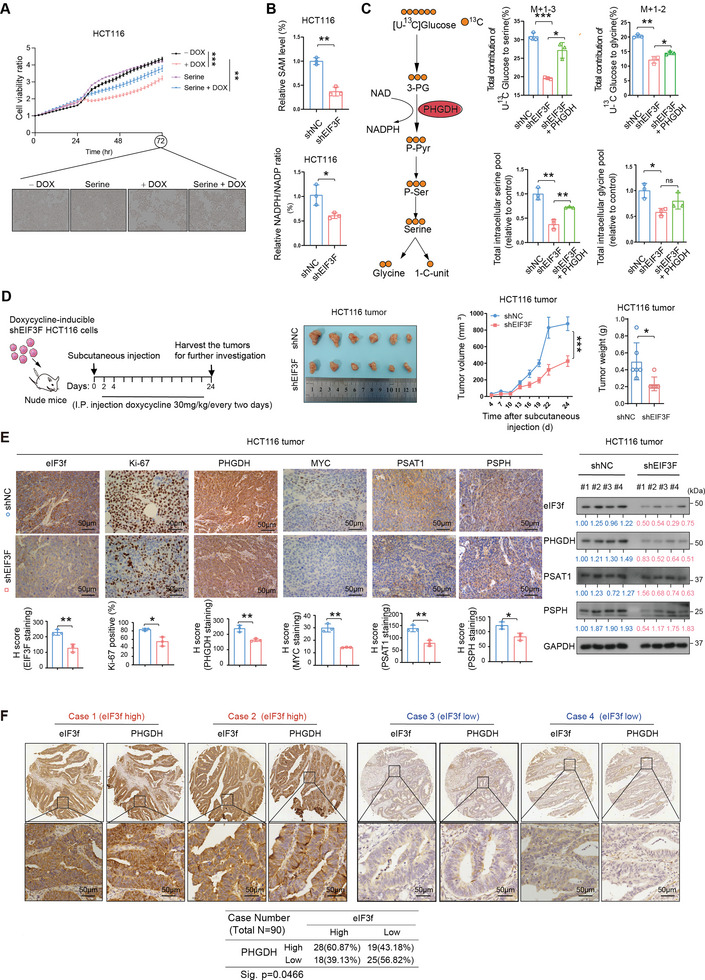
eIF3f regulates SGOC pathway in CRC. A) Serine could partially rescue the growth inhibition of CRC cells induced by EIF3F knockdown in HCT‐116 cells. IncuCyte was used to measure the confluence of the cells. Each time point was relative to Time 0 h. TWO WAY‐ANOVA test was used to test the significance. ** *P* <0.01, ***P < 0.001. B) Measurement of SAM and NADH/NAD+ levels in HCT‐116 cells transduced with EIF3F shRNA. *P < 0.05, **P < 0.01. C) Scheme of metabolite tracing of [U‐^13^C]‐labeled glucose to serine and glycine metabolism (Left). Incorporation of [U‐^13^C] glucose into the indicated metabolites at 24 h in HCT‐116 cells expressing indicated plasmids (Right). *P < 0.05, **P < 0.01, *** *P* <0.001. D) Tumor growth curves of HCT‐116 (1×10^6^) colon cancer cells with or without EIF3F knockdown. EIF3F knockdown was induced by doxycycline(30 mg kg^−1^) treatment. Cells were subcutaneously injected into nude mice (*n* = 6). The tumors were isolated at the end of the experiments. Tumor volume and tumor weight were measured. E) Representative IHC images of EIF3F, Ki67, PHGDH, MYC, PSAT1, and PSPH staining in the subcutaneous tumor tissues generated in (H). Staining intensity were quantitated. Unpaired student's *t* test was used to test the significance, *P < 0.05, **P < 0.01. Scale bars represent 50 µm. Immunoblot analysis of indicated protein levels in the subcutaneous tumor tissues generated in (H). F) Representative EIF3F and PHGDH IHC staining in the tissue microarray (TMA). Case 1 and 2 are representatives of a patient with EIF3F high‐expressed colon cancer. Case 3 and 4 are representatives of a patient with EIF3F low‐expressed colon cancer. Chi‐square analysis shows the correlation of eIF3f and PHGDH expression in human CRC tissue microarray specimens (*n* = 90).

The data in this study suggest that eIF3f positively regulated PHGDH expression, thus influencing the SGOC pathway. Indeed, eIF3f KD exhibited growth suppression, while serine supplement^[^ ^26^ ^]^ could rescue the decreased cell viability caused by eIF3f KD (**Figure** **5**A and Figure S5A, Supporting Information). In one‐carbon metabolism pathway, S‐adenosylmethionine (SAM) and NADPH are critical for tumor‐initiating cells. We found that the SAM level and NADPH/NADP+ ratio were decreased in the eIF3f KD cells (Figure 5B). Further, we performed [U‐^13^C]‐glucose tracing experiment to detect whether the SSP pathway‐related metabolites were changed via eIF3f‐PHGDH axis (Figure 5C) and the eIF3f KD or PHGDH OE efficiency were confirmed by immunoblotting (Figure S5B, Supporting Information). The results showed that eIF3f KD could decrease the amount of serine and glycine, while ectopic‐expression of PHGDH could partially revert this phenomenon in the presence of eIF3f KD (Figure 5C).

To further explore the tumorigenic capacity of eIF3f in CRC cells, we used CRC xenograft model in nude mice subcutaneously injected with HCT‐116 cells expressing pLKO‐Tet‐on‐Doxycycline‐inducible sh‐eIF3f. The result showed that using doxycycline inducing sh‐eIF3f, knockdown of eIF3f suppressed tumor growth in vivo (Figure 5D). Immunohistochemistry and immunoblotting showed that tumors with eIF3f KD exhibited a marked reduction in proliferation marker Ki‐67 and reduced the PHGDH, PSPH, PSAT1, and MYC expression (Figure 5E). Further, we implanted fresh primary CRC tumor samples resected from CRC patients into the immunocompromised mice to establish patient‐derived xenografts (PDX). The eIF3f levels were measured in these PDX tumors (Figure S5C, Supporting Information). Significantly, administration of the PHGDH inhibitor NCT‐503 in the established eIF3f high PDX tumors (CRC#116, 216) attenuated tumor growth; while NCT‐503 had little impact on the growth of eIF3f low PDX tumors (CRC#309, 490) (Figure S5D and S5E, Supporting Information). Tunel staining indicated that NCT‐503 treatment could induce more apoptotic signal in the eIF3f high PDX tumors but not in the eIF3f low PDX tumors (Figure S5F, Supporting Information).

Significantly, human CRC tissue microarrays analysis revealed that eIF3f expression positively correlates with PHGDH expression based on Immunohistochemistry staining (Figure 5F). Collectively, these results validated the oncogenic role of eIF3f and that eIF3f promoted CRC progression via SGOC pathway reprogramming by regulating PHGDH expression.

### 2.6 Wnt Signaling Transcriptionally Upregulates the Expression of eIF3f

**Figure 6:**
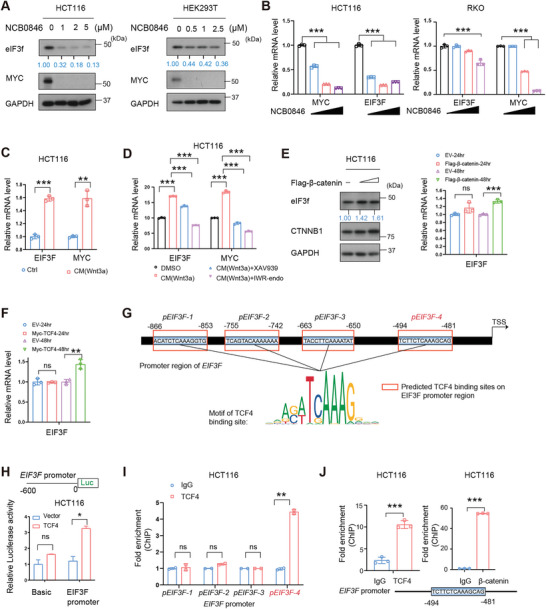
Wnt signaling promoted EIF3F transcription. A) Immunoblotting of EIF3F in HCT‐116 (left panel) and HEK293T (right panel) cells treated with the indicated concentrations of WNT inhibitor (NCB0846) for 48 h. MYC is measured as a positive control of NCB0846 treatment. B) qRT‐PCR analysis of EIF3F in indicated cells treated with the indicated concentrations of WNT inhibitor (NCB0846) for 48 h. Student's *t*‐test was used. *** *P* < 0.001. C) qRT‐PCR analysis of EIF3F in HCT‐116 cells treated with Wnt‐3a containing conditioned medium for 12 h. Student's *t*‐test was used. D) qRT‐PCR analysis of EIF3F in HCT‐116 cells treated with indicated treatment. Student's *t*‐test was used. *** *P* <0.001. E) Immunoblotting results and qRT‐PCR analysis of EIF3F expression in HCT116 with β‐catenin overexpression. *** *P* < 0.001, ns = no significant. F) qRT‐PCR analysis of EIF3F expression in HCT116 cells expressing TCF4. **P < 0.01, ns = no significant. G) Four potential TCF4 binding sites in the promoter of EIF3F predicted by JASPAR website. H) Luciferase activity was detected by dual luciferase reporter assay after HEK‐293T cells transfected with the indicated reporter plasmids (Basic or EIF3F promoter) and TCF4 expression plasmids. *P < 0.05, ns = no significant. I) Chromatin immunoprecipitation of TCF4 and IgG in HCT116 cells, followed by qPCR for the indicated loci on *EIF3F* promoter. Data were presented as mean ± SD of three independent experiments. **P < 0.01, ns = no significant. J) TCF4 and β‐catenin binds to the same site of EIF3F promoter. ChIP analysis of EIF3F promoter in HCT‐116 cells using antibodies against TCF4 (left panel) and β‐catenin (right panel). Bars represent means ± SD, *n* = 3, student's *t* test. *** *P* <0.001.

As *EIF3F* gene is elevated in CRC, we sought to identify the potential transcription factor that is involved in *EIF3F* transcription regulation. Using JASPAR website (https://jaspar.genereg.net/) to search on potential transcription factors of *EIF3F*, we found several potential binding sites of transcription factor TCF7L2 (or TCF4) in the *EIF3F* promoter region, indicating that *EIF3F* might be transcriptionally regulated by Wnt signaling pathway. Given that Wnt signaling is highly activated in CRC progression, we further treated CRC cells with Wnt signaling inhibitor, NCB0846, and the immunoblotting and qRT‐PCR results showed that NCB0846 treatment reduced eIF3f expression in dose‐dependent manners (**Figure** **6**A,B). Congruently, activation of Wnt signaling pathway by Wnt‐3A promoted eIF3f expression (Figure 6C,D). Furthermore, expression of Wnt signaling mediator β‐catenin/TCF4 indeed upregulated eIF3f gene expression (Figure 6E,F). By searching for the TCF4 consensus sequence in details, we identified the *EIF3F* promoter containing the TCF4 binding sites located between −886 and −481 (Figure 6G). By linking the *EIF3F* promoter region to a luciferase reporter, we found that TCF4 overexpression upregulated *EIF3F* luciferase reporter gene activity (Figure 6H). TCF4 ChIP analysis showed that TCF4 bound to this TCF4 binding motif (−494 to −481) on *EIF3F* promoter while the other binding sites are not affected (Figure 6I). Further we identified the −494CACAGCTGCG‐484 motif on *EIF3F* promoter as the binding site of the β‐catenin/TCF4 complex as the ChIP assays revealed that both TCF4 and β‐catenin bind to it (Figure 6J). These data indicate that *EIF3F* is a transcriptional target of the Wnt signaling pathway via β‐catenin/TCF4 binding to the *EIF3F* promoter.

### 2.7 Combination Treatment of NCT503 and LGK‐974 Mitigated Tumor Growth in eIF3f High Human PDX CRC Models with Better Efficacy through Hindering Wnt‐eIF3f‐PHGDH Signaling Axis

**Figure 7:**
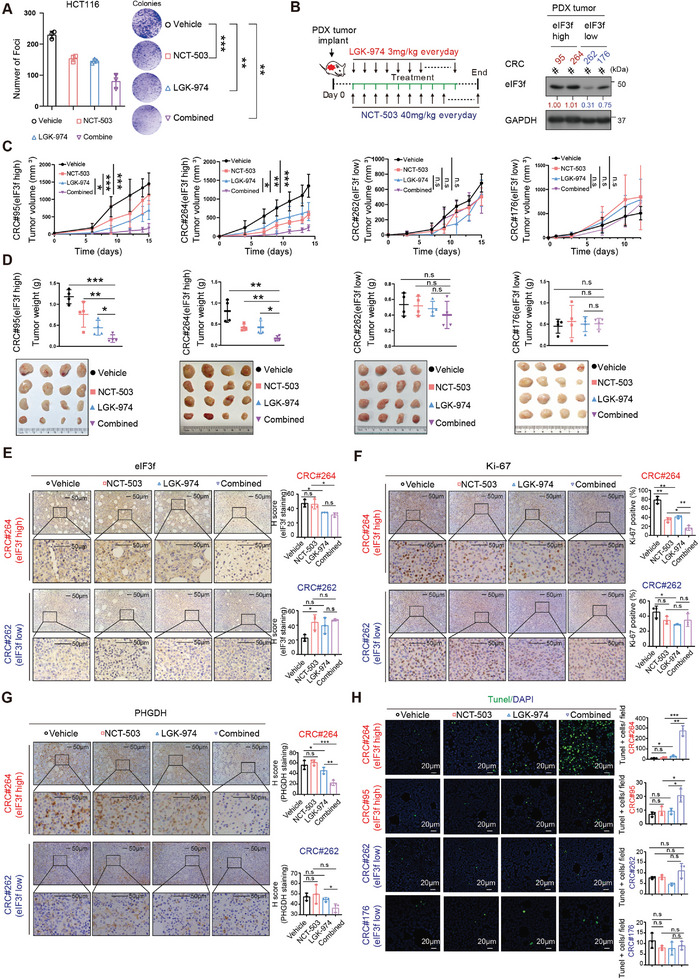
Combined treatment of NCT‐503 and LGK‐974 reduced EIF3F high CRC PDX tumors growth with better efficacy in vivo. A) Quantitation and representative images showed the inhibitory effect on HCT‐116 colony formation by different treatment. B) The scheme of combined treatment on CRC PDX model (Left). Treatment schedule of PHGDH inhibitor NCT‐503 and Wnt inhibitor LGK‐974 were indicated. The mice were treated with indicated treatment via intraperitoneal injection. Immunoblotting of the expression level of EIF3F in indicated CRC PDX tumors was shown (Right). C,D) Tumor volume and tumor weight of EIF3F high CRC PDX tumor or EIF3F low CRC PDX tumor with indicated treatment. Xenograft PDX tumor volume was measured twice a week. E–G) Representative images of immunohistochemical staining were used to determine the expression of Ki‐67, EIF3F, and PHGDH in indicated CRC PDX tumors following indicated treatment. H) Combined treatment of NCT‐503 and LGK‐974 significantly induced apoptosis in eIF3f high CRC PDX tumor. Representative immunofluorescent images and quantitation of apoptotic TUNEL^+^ tumor cells in all CRC PDXs after indicated treatment were shown. *P < 0.05, **P < 0.01, *** *P* <0.001, ns = no significant.

To validate the relevance of our findings to human CRC and to examine whether hindering eIF3f‐PHGDH signaling axis can restrain the CRC tumor formation, we performed cell growth studies and the results showed that both NCT‐503 and LGK‐974 (Wnt inhibitor) could suppress CRC foci formation. The combination of NCT‐503 and LGK‐974 was more efficient in inhibiting CRC foci formation than NCT‐503 or the LGK‐974 alone (**Figure** **7**A). Next, we established PDX model^[^ ^27^ ^]^ for testing the drug efficacy of combined treatment on CRC tumor growth in two CRC PDX sets (eIF3f high versus eIF3f low), and the expressing levels of eIf3f were characterized by immunoblotting (Figure 7B). The combination of NCT‐503 and LGK‐974 was more efficient in suppressing tumor growth than NCT‐503 or LGK‐974 alone in the eIF3f high‐expressing PDX tumors, while the efficacy of these drugs on the eIF3f low‐expressing PDX tumors was compromised in terms of tumor volume and weights (Figure 7A,D). Congruently, the administration of the combination of NCT‐503 and LGK‐974 in eIF3f‐high group dramatically diminished the expression of Ki67, and increased the Tunel signals; while the impact on eIF3f‐low group is less effective (Figure 7E–H). Together, targeting both Wnt signaling and PHGDH activity may be considered for therapeutic strategy for eIF3f high CRC patients.

## 3 Discussion

**Figure 8:**
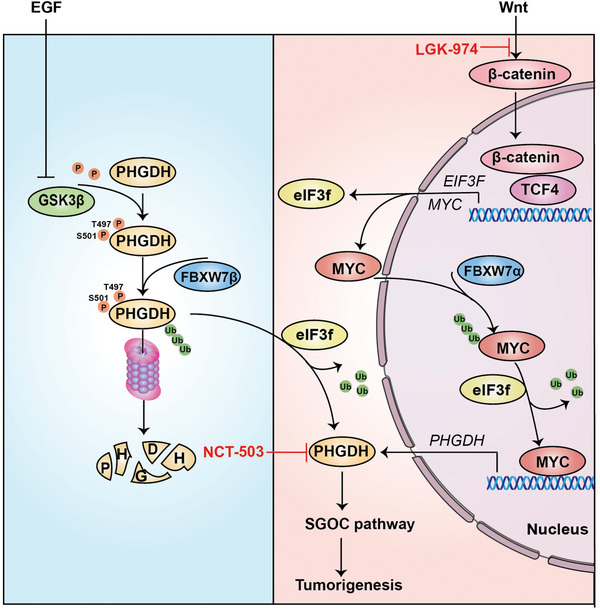
Schematic summary of eIF3f's deubiquitinating role in regulating PHGDH expression. Model depicts that upon activation of the EGFR‐GSK3β axis, PHGDH is not vulnerable to FBXW7β‐mediated ubiquitination, and is thus stabilized. On the other hand, high eIF3f has a positive impact on PHGDH stability as eIF3f antagonizes Fbxw7β‐mediated PHGDH ubiquitination through its deubiquitinating activity even when EGF is not present. In response to Wnt signaling, β‐catenin/TCF4 directly binds to the promoter of *EIF3F* to enhance eIF3f transcription. Further, Wnt‐elevated MYC, which is deubiquitinated by eIF3f, can in turn facilitates transcriptional expression of PHGDH to enhance the SGOC pathway, thereby facilitating tumorigenesis.

eIF3f, previously known as one of the subunits of the translation initiation factor eIF3, is a protein that functions not only in translation initiation but also in gene regulation,^[^ ^28^ ^]^ and diseases.^[^ ^29^ ^]^ It actually links to ubiquitin‐mediated protein degradation machineries,^[^ ^30^ ^]^ to regulate important targets involved in cancers. However, the upstream regulators and downstream targets of eIF3f has not been fully characterized. Here we characterize that Wnt pathways have activities in regulating eIF3f expression through β‐catenin and TCF4 signaling and eIF3f is overexpressed in CRC. Furthermore, eIF3f acts as a deubiquitinating enzyme regulating PHGDH post‐transcriptionally depending on EGF‐GSK3β signaling. Moreover, eIF3f can deubiquitinate and stabilize MYC, a Wnt target and a transcription activator of PHGDH, to regulate PHGDH transcriptionally. Our data shed light on eIF3f upstream regulatory circuit and reveal how EGF/Wnt oncogenic signal in promoting eIF3f‐PHGDH axis to enhance the SGOC pathway, thereby affecting tumorigenesis (**Figure** **8**).

However, the regulations and biological activities of eIF3f were not fully characterized in cancers. Our data fill this knowledge gap by identifying that eIF3f is highly expressed in CRC, and by characterizing its oncogenic activities including impacts on cell proliferation, metabolism, and tumorigenesis. However, there is a discrepancy regarding eIF3f's role in cancer. Studies showed that high expression of eIF3f correlated with advanced gastric tumor stages and likelihood of recurrence^[^ ^31^ ^]^ just like CRC, while eIF3f expression is significantly decreased in human pancreatic adenocarcinoma and melanoma,^[^ ^32^ ^]^ and eIF3f inhibits tumor growth in cervical cancer model.^[^ ^33^ ^]^ Yet, it is not clear how this discrepancy occurs. It remains to be investigated whether the targets of eIF3f determine the functional outcome of eIF3f expression in various types of cancers and how the context‐dependent expression level of eIF3f occurs.

Dysregulation of PHGDH, the key enzyme in serine synthesis, plays an important role in a variety of cancers.^[^ ^34^ ^]^ PHGDH overexpression is involved in migration, drug resistance,^[^ ^35^ ^]^ and metastasis.^[^ ^36^ ^]^ Recent studies demonstrate that PHGDH ubiquitination is regulated by Parkin, an E3 ubiquitin ligase involved in Parkinson's disease, in human breast cancer and lung cancer,^[^ ^37^ ^]^ RNF5 in breast cancer,^[^ ^38^ ^]^ and RNF114 in liver or kidney.^[^ ^38^ ^]^ Thus, three additional different E3 ligases can participate in PHGDH stability regulation. Our results show that GSK3β mediates PHGDH phosphorylation, which destabilizes PHGDH through enhancing FBXW7β‐mediated PHGDH ubiquitination. Meanwhile, eIF3f has an intrinsic deubiquitinating enzyme activity to deubiquitinate FBXW7β‐mediated PHGDH ubiquitination. It remains to be determined whether these regulations are context‐ or ‐tissue specific and are antagonized by eIF3f as well. Interestingly, eIF3f also deubiquitinate FBXW7α‐mediated MYC ubiquitination. Thus, eIF3f has impacts on both MYC and PHGDH, which in turn elicits a plethora of oncogenic signals.^[^ ^39^ ^]^ Our study identified PHGDH as a novel substrate of FBXW7β and implies that eIF3f may participate in deubiquitinating FBXW7 targets.

Wnt signaling is a pivotal oncogenic pathway in CRC, and targeting this pathway for therapeutic strategy is very appealing. LGK974, a potent and specific small molecule Porcupine (PORCN) inhibitor, has been demonstrated to block WNT signaling and to be used in phase I clinical trial.^[^ ^40^ ^]^ Our study demonstrated that LGK974 potently reduced the expression of eIF3f by inhibiting Wnt signaling. Given that Wnt signaling pathway‐induced eIF3f positively regulate PHGDH activity, it lends credence to the possibility that targeting PHGDH activation (NCT503) plus Wnt signaling (LGK974) might have a better synergistic effect in treating eIF3f‐high CRC. Indeed, our study demonstrated that a combination treatment of LGK974 and NCT503 leads to the high response rate in inhibiting eIF3f‐high CRC PDX model, which provided strong support for targeting eIF3f‐driven CRC through the combination treatment as a novel therapeutic strategy for CRC therapy.

In summary, the present study demonstrates that eIF3f is a critical oncogenic factor and provides strong evidence for eIF3f‐PHGDH axis as a novel therapeutic target for CRC patients. Furthermore, eIF3f exerts its oncogenic function by deubiquitinating PHGDH and MYC, which subsequently enhances SGOC pathway to promote CRC progression. Moreover, we uncover that PHGDH is a novel substrate of GSK3β/FBXW7β and eIF3f could deubiquitinate FBXW7β‐mediated ubiquitination of PHGDH. Thus, therapeutic approaches targeting eIF3f‐PHGDH axis represent an attractive strategy for eIF3f‐overexpressing CRC patients and further developing compounds that suppress eIF3f‐mediated PHGDH stabilization and inhibit Wnt signals are worthy of more investigation.

## 4 Experimental Section

### Human Samples

26 Fresh frozen paired samples of primary colorectal cancer and adjacent normal colon tissue were collected from the Department of Surgery at the Sixth Affiliated Hospital of Sun Yat‐sen University. All samples were collected with the patients’ written informed consent and approval from study center's Institutional Review Board. The paraffin‐embedded colorectal cancer tissue microarray (TMA) was obtained Outdo Biotech (Shanghai Outdo biotech co., Ltd.). The original immunohistochemistry slides were scanned by Slide Scanning System SQS‐1000 (TEKSQRAY). TMA images were analyzed with HALO image analysis software (Indica Labs).

### Published Datasets and Analysis

Colon cancer tissue (COAD) expression profiles of TCGA were downloaded from UCSC Xena (http://xena.ucsc.edu/). GSE77953, GSE9348, GSE71187, and GSE41258 expression profiles were downloaded from the Gene Expression Omnibus database. When published datasets were assessed by GSEA, the Pearson metric was used for ranking genes and the phenotype permutation type was selected; for all other parameters, default settings were used.

### Cell Culture, Reagents, and Transfection

All the cells were obtained from ATCC, and maintained at 37 °C and 5% CO_2_ (Thermo, Waltham, MA, USA). HCT‐116, HCT8, HCT15, and DLD1 cells were maintained in RPMI 1640 medium (RPMI). HEK293T, NCM460, HT29, WiDR, SW480, and SW620 cells were maintained in Dulbecco's modified Eagle's medium media (DMEM). RKO and WiDr cells were maintained in ATCC‐formulated Eagle's Minimum Essential Medium (MEM). All the cell culture medium was supplemented with 10% (v/v) fetal bovine serum (FBS), 100 U mL^−1^ penicillin, 100 mg mL^−1^ streptomycin, and 2 × 10^−3^ m l‐glutamine. All transient transfections of plasmids into cell lines followed the standard protocol for PEI MAX 40 000 (Polysciences Inc, #24765‐1).

### Plasmids and Doxycycline Inducible Cells Construction

EIF3F cDNA and PHGDH cDNA were amplified by PCR from HEK293T cells and cloned into PCMV5 vector and PCDNA3.1 with Flag or MYC tag. Mutants of EIF3F were generated by using a Fast Mutagenesis Kit V2 (Vazyme) and further verified by sequencing. For doxycycline‐inducible EIF3F knock down, shRNAs were inserted into Tet‐on‐PLKO vector as sequence listed in Table S1 (Supporting Information). For lentivirus preparation, HEK293T cells were seeded in a 10 cm dish at a density around 1 × 10^6^, and were cotransfected with 10 µg indicated shRNA lentiviral plasmid, 5 µg psPAX2 and 5 µg pMD2.G by using polyethylenimine (Polysciences, 24765). The supernatant which contained lentivirus was collected at 48 and 72 h after transfection, and were filtered through Millex‐GP Filter Unit (0.22 µm pore size, Millipore). Cells were infected with filtered viral supernatant containing 10 µg mL^−1^ polybrene (Millipore, TR‐1003‐G), followed by puromycin selection and finally verified by western blot. PCDNA3.1‐Flag‐FBXW7 was kept in the lab.

### mRNA Expression Analysis

Total RNA was extracted from cells by TRIzol Reagent (Invitrogen, #15596026), and reverse‐transcribed to cDNA by ReverTra Ace qPCR RT Master Mix (TOYOBO). Quantitative PCR (qPCR) was carried out using 2× SYBR Green qPCR Master Mix ((biotool, #B21203) in a LightCycler 480 II instrument (Roche). All the genes expression were normalized to *ACTIN*. The qRT‐PCR was performed by analyzing samples in triplicate. The sequences of the primers for qRT‐PCR are listed in Table S2 (Supporting Information).

### Immunoblotting

Cells or tissues were lysed with cell lysis buffer (50 × 10^−3^ m Tris–HCl PH 7.5, 150  × 10^−3^ m NaCl, 1  × 10^−3^ m EDTA, 1% NP‐40) containing protease inhibitors cocktail and phosphatase inhibitors (Bimake, B15002/B14002). The collected proteins were separated by sodium dodecyl sulfate‐polyacrylamide gel electrophoresis and then the proteins were transferred to polyvinylidene difluoride membranes (Millipore, #IPVH00010). The membranes were blocked with 5% slim milk (Sangon Biotech, #A600669‐0250) for 1 h at room temperature followed by incubation with indicated primary antibodies. Subsequently membranes were washed in Tris‐buffered saline with Tween‐20 (Sangon Biotech, #A600560‐0500) and incubated for 1 hour at room temperature with indicated peroxidase‐conjugated secondary antibodies (Thermo Scientific, #31430). After that, membranes were wash with several times and the chemiluminescent images of immunodetected bands on the membranes were obtained on the X‐ray films (Fujifilm, SUPER RX‐N‐C) using the enhanced chemiluminescence (ECL) system (Bio Rad, #170‐5061. The primary antibodies and dilution ratio were listed in Table S3 in the Supporting Information.

### Immunohistochemistry

Briefly, paraffin‐embedded CRC tumor sections (3 µm) were first deparaffinized and then boiled in citrate buffer (pH 6.0) for antigen retrieval, followed by hydrogen peroxide/PBS blocking of endogenous peroxidase. Slides were preblocked and then probed with indicated antibodies listed in Table S3 in the Supporting Information at 4 °C overnight. The slides were then washed with PBS and incubated with secondary antibodies at room temperature for 15 min and stained with DAB substrate. The cell nuclei were stained with hematoxylin. Quantification of the percentages of indicated antibodies per area were performed using ImageJ. Three fields per tumor were chosen for quantification.

### In Situ Proximity Ligation Assay (PLA)

PLA was performed as previously described.^[^ ^41^ ^]^ Briefly, PLA was performed according to the standard commercial protocol (Sigma‐Aldrich, DUO92101). Briefly, HCT‐116 cells were washed with PBS and then fixed with 4% PFA for 10 min at room temperature. After that, fixed HCT‐116 were permeabilized with PBS containing 0.5% Triton X‐100 for 10 min. Next, cells were blocked with the PLA blocking solution (Duolink II) for 1 hour followed by the incubation of the primary antibodies. All samples were kept in a wet chamber and incubated for overnight at 4 °C. Subsequently, appropriate PLA secondary probe solution was added to the samples and they were incubated at 37 °C for 1 h. Ligation mix was then applied to each of the sample to complete the ligation process at 37 °C for 30 min. Samples were then incubated with polymerization mix for the amplification and incubated at 37 °C for 100 min. Following the incubation, samples were washed once with 1× buffer B for 10 min at room temperature. This was followed by one further wash with 0.01× buffer B for 1 minute. Then samples were mounted using Duolink in situ mounting medium with DAPI for 15 minutes and the PL signal was imaged using confocal microscope.

### Coimmunoprecipitation (Co‐IP)

After indicated treatment, cells were lysed with cell lysis buffer (50  × 10^−3^ m Tris–HCl PH 7.5, 150  × 10^−3^ m NaCl, 1  × 10^−3^ m EDTA, 1% NP‐40) containing protease inhibitors cocktail and phosphatase inhibitors (Bimake, B15002/B14002). For each lysate, supernatants were collected after centrifugation and incubated with appropriate antibodies overnight at 4 °C, followed by protein A/G beads incubation for 4 h (Santa Cruz Biotechnology, CA, USA, #sc‐2002). For IP of Flag‐tagged proteins, anti‐Flag M2 affinity gel (Sigma‐Aldrich, #A2220) was used. After incubation, beads were washed with cell lysis buffer for three times. After that, add propriate volume of 2× loading buffer to the beads and boiled for 15 minutes in 95 °C to get the eluted proteins. Next, immunoblot assays were performed with specific antibodies. The antibodies used for Co‐IP or immunoblot assay were listed in Table S3 in the Supporting Information.

### Ubiquitination Assay

For ubiquitination assay, method was used as previously described.^[^ ^41^, ^42^ ^]^ Briefly, cells were transfected with the indicated plasmids. After 42 h, cells were treated with (10–50) × 10^−6^ MG132 for 6 h. Then the cell lysates were harvested in denaturing buffer (6 m guanidine·HCl, 10 × 10^−3^ m imidazole, 0.1 m Na2HPO4/NaH2PO4; pH 8.0). Add 50 µL nickel beads to the cell lysates and then incubate overnight at 4 °C with rotation. Wash and elute the protein complexes for western blot analysis.

### Turnover Assay

Protein turnover was performed as previously described.^[^ ^43^ ^]^ Briefly, cells were treated with 100 µg mL^−1^ cycloheximide and harvested at indicated time points after cycloheximide treatment. The protein levels were analyzed by western blotting.

### Cell lysates Fractionated by Gel Filtration

HCT‐116 cells expressing PLKO‐tet‐on‐shEIF3F were treated with doxycycline or no treatment for 72 hours. Cells were lysed in lysis buffer (50  × 10^−3^ m Tris pH 7.5, 0.5% Nonidet P‐40, 0.1% Triton X‐100, 150  × 10^−3^ m NaCl, 0.1 m EDTA). Approximately 5 mg protein was concentrated to 0.5 mL Superose 6 Increase 10/300 GL (Cytiva, 29091596) gel filtration column was equilibrated and eluted with the cold PBS buffer (0.01 m phosphate buffer, 0.14 m NaCl, pH 7.4). Cell lysates were loaded into the column and fractionated through the column equipped with Cytiva chromatography system at a flow rate of 0.4 ml/min. Eluants were collected 300 µL/fraction followed by boiling at 95 °C for 10 min. Boiled fractions were resolved by SDS‐PAGE and immunoblotted with indicated antibodies.

### [U‐^13^C]‐Labeled Glucose Tracing Experiment

Metabolite tracing was performed as previously described.^[^ ^42^ ^]^ Briefly, HCT‐116 cells were treated with indicated treatment. After that, cells were removed the cultured medium and cultured in glucose free RPMI‐1640 medium, supplemented with 11  × 10^−3^ m [U‐^13^C]‐labeled glucose. After 24 h, cells were washed twice with cold PBS and extracted with a mixture solvent containing acetonitrile, water and formic acid (80:19:1, v/v/v). Cells were scraped, subjected to two freeze‐thaw cycles and centrifuged for 5 min at 13 000 rpm. 5 µL, 0.03 mg mL^−1^ internal standard, 4Cl‐phenylalanine, was added to the precipitate and then re‐extracted with methanol, and supernatants were pooled in a tube for evaporation under N2 evaporator.

### S‐Adenosyl Methionine (SAM) Determination

SAM levels of CRC cells with or without EIF3F knockdown were measured by using a SAM fluorescence assay kit (Mediomics, #FM‐75‐506). Briefly, cells were lysed with buffer CM and incubated at 24 °C for 1 h, with occasional vortex followed by centrifugation to collect the supernatant for SAM assay determination. To measure the SAM level, fluorescence signal intensity was read using a fluorescence microplate reader (excitation ≈ 485 nm, emission ≈ 665 nm).

### NADPH/NADP Determination

NADPH/NADP determination was performed using NADP+/NADPH Assay Kit (Beyotime Biotechnology, #S0179) with WST‐8 according to manufacturer's instructions.

### RNA Seq Analysis

RNA‐Seq analysis was performed in HCT‐116 cells with or without eIF3f knockdown. Total RNA extracted from the indicated groups of HCT‐116 cells was subjected to RNA sequencing (RNA‐Seq) performed by SHANGHAI BIOTECHNOLOGY CORPORATION (Shanghai, China). The sequencing reads were analyzed to obtain expression profiles. Gene set enrichment analysis (GSEA) used the GSEA software provided by the Broad Institute (http://www.broadinstitute.org/gsea/index. jsp), in accordance with the instructions provided by the Broad Institute. Hallmark gene sets were used within the Molecular Signatures Database version 7.0.

### Mouse Models

All mice were purchased from Model Animal Research Center of Nanjing University (Nanjing, China). All animals were maintained under standard laboratory conditions, with free access to food and water. All animal experiments were performed under protocols approved by the Institutional Animal Care and Use Committee of The Sixth Affiliated Hospital of Sun Yat‐sen University (NO.20181123).

For Colorectal cancer Xenograft model, eight‐week‐old female nude mice were randomly grouped and subcutaneously injected with 1×10^6^ HCT116 cells, containing stably tetracycline‐inducible shEIF3F and were treated with either PBS (control group) or 50 mg kg^−1^ doxycycline (eIF3f KD group) via intraperitoneal injection. Tumor volumes were measured and recorded. At the end of the experiment, mice were sacrificed by CO_2_ inhalation and the tumors were harvested and weighted, and collected for further analysis.

For Patient‐derived xenograft (PDX) model, implantation of PDXs was conducted as described before^[^ ^42^ ^]^. Briefly, Patient‐derived tumor fragments (3‐4mm^3^) were subcutaneously implanted bilaterally into the flank of 6‐ to 8‐week‐old age female NSG mice under general anesthesia. Mice were assigned randomly into indicated treatment groups when tumors reached ≈50 mm^3^.

### Luciferase Reporter Assay

Luciferase reporter assay was performed as previously described.^[^ ^44^ ^]^ Basically, cells seeded in 24‐well plates were transfected with indicated amount of pGL3 or pGL3‐pEIF3F containing a TCF7L2 transcription factor binding site on EIF3F, pRL‐CMV, and pCMV‐MYC‐TCF7L2 expressing plasmid into HEK293T cells. After 48 h, cells were lysed, and the reporter activity was assayed with the dual luciferase assay system (Promega, #E1960) according to the manufacturer's instructions.

### ChIP

ChIP assays were performed using EZ‐ChIP Kits (Millipore, #17‐10085) according to the protocol described^[^ ^41^ ^]^. Briefly, cells were treated as indicated treatment. After 48–72 h, cells were crosslinked for 10 min using 1% PFA and were then quenched with glycine. Cells were then lysed in cell lysis buffer to break the cell membrane. Then centrifuge the lysate and resuspended in nuclear lysis buffer and sonicated. After centrifugation, the supernatants were diluted as protocol required and incubated with 1 µg aliquots of primary antibodies (anti‐TCF7L2, control mouse IgG, or anti‐cMYC, control rabbit IgG,) and ChIP beads overnight. Antibody bound protein/DNA complexes were then pulled down using ChIP beads and were washed with Low salt buffer, high salt buffer and LiCl buffer, TE buffer. After that, each ChIP were eluted with elution buffer. Eluted protein/DNA complexes were finally digested with protease K and purified DNA samples were analyzed by qRT‐PCR for TCF7L2 binding sites or MYC binding sites with the primer sets listed in Table S2 (Supporting Information).

### TUNEL Staining

Apoptotic cells in CRC tumor tissues were measured by TUNEL Apoptosis Assay Kits. Briefly, paraffin‐embedded CRC tumor tissue sections were pretreated by proteinase K for 20 min at room temperature. Then tissue sections were washed three times with PBS and then incubated in the mixture of reaction buffer with TdT enzyme in dark for 60 min at 37 °C followed by the cell nucleuses staining with DAPI. The tissue sections were observed and photographed using the fluorescence microscope (LSM 880 with Fast Airyscan).

### Statistical Analysis

Student's *t* test or Mann–Whitney tests were performed to evaluate differences between two or multiple groups. Kaplan–Meier survival analysis was used to analyze the patient survival. The Pearson's correlation between two proteins expression was calculated using GraphPad statistics software (GraphPad Software version 7, La Jolla, CA, USA). Data are presented as the mean ± SD of three independent experiments. For all analyses, *p* < 0.05 was considered to statistical significance.

## Conflict of Interest

The authors declare no conflict of interest.

## Author Contributions

Q.P., F.Y., and H.J. contributed equally to this work. Conceptualization: M.‐H.L., X.M.; Sample collection and processing: Q.P., X.M., H.J., R.L., H.Y.; Methodology: Q.P., X.M., M.‐H.L.; Investigation: Q.P., F.Y., X.H., P.Z., J.P., X.X., X.L., N.M., Y.W., J.Z., B.Y., W.W.; TMA analysis: Q.P., X.X.; Bioinformatics analysis and statistics: Q.P., X.M. Supervision: M.‐H.L. and X.M. Manuscript preparation and writing: Q.P., X.M., M.‐H.L. All authors reviewed and approved the manuscript for publication.

## Data and Materials Availability

All data needed to evaluate the conclusions in the paper are present in the paper and/or the Supplementary Materials.

## Supporting information

Supporting Information

## Data Availability

The data that support the findings of this study are available from the corresponding author upon reasonable request.

## References

[1] a) R. L. Siegel , K. D. Miller , H. E. Fuchs , A. Jemal , CA ‐ Cancer J. Clin. 2021, 71, 7; 33433946 10.3322/caac.21654

[2] M. S. Reimers , E. C. Zeestraten , P. J. Kuppen , G. J. Liefers , C. J. van de Velde , Gastroenterol. Rep. 2013, 1, 166. 10.1093/gastro/got022 PMC3937997 24759962

[3] a) S. Zou , L. Fang , M. H. Lee , Gastroenterol. Rep. 2018, 6, 1; 10.1093/gastro/gox031 PMC5806407 29479437

[4] a) D. Crosby , S. Bhatia , K. M. Brindle , L. M. Coussens , C. Dive , M. Emberton , S. Esener , R. C. Fitzgerald , S. S. Gambhir , P. Kuhn , T. R. Rebbeck , S. Balasubramanian , Science 2022, 375, eaay9040; 35298272 10.1126/science.aay9040

[5] A. Herrmannová , D. Daujotyte , J. C. Yang , L. Cuchalová , F. Gorrec , S. Wagner , I. Dányi , P. J. Lukavsky , L. S. Valásek , Nucleic Acids Res. 2012, 40, 2294. 22090426 10.1093/nar/gkr765 PMC3300007

[6] R. Marchione , S. A. Leibovitch , J. L. Lenormand , Cell. Mol. Life Sci. 2013, 70, 3603. 23354061 10.1007/s00018-013-1263-y PMC3771369

[7] A. des Georges , V. Dhote , L. Kuhn , C. U. Hellen , T. V. Pestova , J. Frank , Y. Hashem , Nature 2015, 525, 491. 26344199 10.1038/nature14891 PMC4719162

[8] a) G. A. Cope , G. S. Suh , L. Aravind , S. E. Schwarz , S. L. Zipursky , E. V. Koonin , R. J. Deshaies , Science 2002, 298, 608; 12183637 10.1126/science.1075901

[9] A. Docquier , L. Pavlin , A. Raibon , C. Bertrand‐Gaday , C. Sar , S. Leibovitch , R. Candau , H. Bernardi , J. Physiol. 2019, 597, 3107. 31026345 10.1113/JP277841

[10] a) Y. Yin , J. Long , Y. Sun , H. Li , E. Jiang , C. Zeng , W. Zhu , Gene 2018, 673, 130; 29908282 10.1016/j.gene.2018.06.034

[11] D. Hanahan , R. A. Weinberg , Cell 2011, 144, 646. 21376230 10.1016/j.cell.2011.02.013

[12] a) M. Mehrmohamadi , X. Liu , A. A. Shestov , J. W. Locasale , Cell Rep. 2014, 9, 1507; 25456139 10.1016/j.celrep.2014.10.026 PMC4317399

[13] T. Muthusamy , T. Cordes , M. K. Handzlik , L. You , E. W. Lim , J. Gengatharan , A. F. M. Pinto , M. G. Badur , M. J. Kolar , M. Wallace , A. Saghatelian , C. M. Metallo , Nature 2020, 586, 790. 32788725 10.1038/s41586-020-2609-x PMC7606299

[14] M. A. Reid , A. E. Allen , S. Liu , M. V. Liberti , P. Liu , X. Liu , Z. Dai , X. Gao , Q. Wang , Y. Liu , L. Lai , J. W. Locasale , Nat. Commun. 2018, 9, 5442. 30575741 10.1038/s41467-018-07868-6 PMC6303315

[15] O. D. Maddocks , C. F. Labuschagne , P. D. Adams , K. H. Vousden , Mol. Cell 2016, 61, 210. 26774282 10.1016/j.molcel.2015.12.014 PMC4728077

[16] a) A. Buqué , L. Galluzzi , D. C. Montrose , Trends Cancer 2021, 7, 668; 34219053 10.1016/j.trecan.2021.06.004 PMC9097339

[17] a) O. D. K. Maddocks , D. Athineos , E. C. Cheung , P. Lee , T. Zhang , N. J. F. van den Broek , G. M. Mackay , C. F. Labuschagne , D. Gay , F. Kruiswijk , J. Blagih , D. F. Vincent , K. J. Campbell , F. Ceteci , O. J. Sansom , K. Blyth , K. H. Vousden , Nature 2017, 544, 372; 28425994 10.1038/nature22056

[18] J. W. Locasale , A. R. Grassian , T. Melman , C. A. Lyssiotis , K. R. Mattaini , A. J. Bass , G. Heffron , C. M. Metallo , T. Muranen , H. Sharfi , A. T. Sasaki , D. Anastasiou , E. Mullarky , N. I. Vokes , M. Sasaki , R. Beroukhim , G. Stephanopoulos , A. H. Ligon , M. Meyerson , A. L. Richardson , L. Chin , G. Wagner , J. M. Asara , J. S. Brugge , L. C. Cantley , M. G. Vander Heiden , Nat. Genet. 2011, 43, 869. 21804546 10.1038/ng.890 PMC3677549

[19] M. Rossi , P. Altea‐Manzano , M. Demicco , G. Doglioni , L. Bornes , M. Fukano , A. Vandekeere , A. M. Cuadros , J. Fernández‐García , C. Riera‐Domingo , C. Jauset , M. Planque , H. F. Alkan , D. Nittner , D. Zuo , L. A. Broadfield , S. Parik , A. A. Pane , F. Rizzollo , G. Rinaldi , T. Zhang , S. T. Teoh , A. B. Aurora , P. Karras , I. Vermeire , D. Broekaert , J. V. Elsen , M. M. L. Knott , M. F. Orth , S. Demeyer , et al., Nature 2022, 605, 747. 35585241 10.1038/s41586-022-04758-2 PMC9888363

[20] R. Zhao , S. C. Yeung , J. Chen , T. Iwakuma , C. H. Su , B. Chen , C. Qu , F. Zhang , Y. T. Chen , Y. L. Lin , D. F. Lee , F. Jin , R. Zhu , T. Shaikenov , D. Sarbassov , A. Sahin , H. Wang , H. Wang , C. C. Lai , F. J. Tsai , G. Lozano , M. H. Lee , J. Clin. Invest. 2011, 121, 851. 21317535 10.1172/JCI44111 PMC3049400

[21] J. S. Lee , L. Adler , H. Karathia , N. Carmel , S. Rabinovich , N. Auslander , R. Keshet , N. Stettner , A. Silberman , L. Agemy , D. Helbling , R. Eilam , Q. Sun , A. Brandis , S. Malitsky , M. Itkin , H. Weiss , S. Pinto , S. Kalaora , R. Levy , E. Barnea , A. Admon , D. Dimmock , N. Stern‐Ginossar , A. Scherz , S. C. S. Nagamani , M. Unda , D. M. Wilson 3rd , R. Elhasid , A. Carracedo , et al., Cell 2018, 174, 1559. 30100185 10.1016/j.cell.2018.07.019 PMC6225773

[22] J. Moretti , P. Chastagner , S. Gastaldello , S. F. Heuss , A. M. Dirac , R. Bernards , M. G. Masucci , A. Israel , C. Brou , PLoS Biol. 2010, 8, e1000545. 21124883 10.1371/journal.pbio.1000545 PMC2990700

[23] C. W. Li , S. O. Lim , W. Xia , H. H. Lee , L. C. Chan , C. W. Kuo , K. H. Khoo , S. S. Chang , J. H. Cha , T. Kim , J. L. Hsu , Y. Wu , J. M. Hsu , H. Yamaguchi , Q. Ding , Y. Wang , J. Yao , C. C. Lee , H. J. Wu , A. A. Sahin , J. P. Allison , D. Yu , G. N. Hortobagyi , M. C. Hung , Nat. Commun. 2016, 7, 12632. 27572267 10.1038/ncomms12632 PMC5013604

[24] W. Wang , J. Li , J. Tan , M. Wang , J. Yang , Z. M. Zhang , C. Li , A. G. Basnakian , H. W. Tang , N. Perrimon , Q. Zhou , Nat. Commun. 2021, 12, 476. 33473107 10.1038/s41467-020-20780-2 PMC7817833

[25] A. D'Avola , N. Legrave , M. Tajan , P. Chakravarty , R. L. Shearer , H. W. King , K. Kluckova , E. C. Cheung , A. J. Clear , A. S. Gunawan , L. Zhang , L. K. James , J. I. MacRae , J. G. Gribben , D. P. Calado , K. H. Vousden , J. C. Riches , J. Clin. Invest. 2022, 132, e153436. 35316216 10.1172/JCI153436 PMC9057607

[26] O. D. Maddocks , C. R. Berkers , S. M. Mason , L. Zheng , K. Blyth , E. Gottlieb , K. H. Vousden , Nature 2013, 493, 542. 23242140 10.1038/nature11743 PMC6485472

[27] A. T. Byrne , D. G. Alférez , F. Amant , D. Annibali , J. Arribas , A. V. Biankin , A. Bruna , E. Budinská , C. Caldas , D. K. Chang , R. B. Clarke , H. Clevers , G. Coukos , V. Dangles‐Marie , S. G. Eckhardt , E. Gonzalez‐Suarez , E. Hermans , M. Hidalgo , M. A. Jarzabek , S. de Jong , J. Jonkers , K. Kemper , L. Lanfrancone , G. M. Mælandsmo , E. Marangoni , J. C. Marine , E. Medico , J. H. Norum , H. G. Palmer , D. S. Peeper , et al., Nat Rev Cancer 2017, 17, 254. 28104906 10.1038/nrc.2016.140

[28] D. Farache , S. P. Antine , A. S. Y. Lee , Trends Cell Biol. 2022, 32, 762. 35466028 10.1016/j.tcb.2022.03.006 PMC9378348

[29] D. A. Wolf , Y. Lin , H. Duan , Y. Cheng , J. Mol. Cell Biol. 2020, 12, 403. 32279082 10.1093/jmcb/mjaa018 PMC7333474

[30] Z. Sha , L. M. Brill , R. Cabrera , O. Kleifeld , J. S. Scheliga , M. H. Glickman , E. C. Chang , D. A. Wolf , Mol. Cell 2009, 36, 141. 19818717 10.1016/j.molcel.2009.09.026 PMC2789680

[31] Y. Cheng , J. Zhou , H. Li , Clin. Transl. Sci. 2015, 8, 320. 25684180 10.1111/cts.12263 PMC5351038

[32] A. Doldan , A. Chandramouli , R. Shanas , A. Bhattacharyya , S. P. Leong , M. A. Nelson , J. Shi , Mol. Carcinog. 2008, 47, 806. 18381585 10.1002/mc.20436 PMC2635928

[33] J. Y. Lee , H. J. Kim , S. B. Rho , S. H. Lee , OncoTargets Ther. 2016, 7, 18541. 10.18632/oncotarget.8105 PMC4951308 26988917

[34] D. C. Montrose , S. Saha , M. Foronda , E. M. McNally , J. Chen , X. K. Zhou , T. Ha , J. Krumsiek , M. Buyukozkan , A. Verma , O. Elemento , R. K. Yantiss , Q. Chen , S. S. Gross , L. Galluzzi , L. E. Dow , A. J. Dannenberg , Cancer Res. 2021, 81, 2275. 33526512 10.1158/0008-5472.CAN-20-1541 PMC8137552

[35] L. Wei , D. Lee , C. T. Law , M. S. Zhang , J. Shen , D. W. Chin , A. Zhang , F. H. Tsang , C. L. Wong , I. O. Ng , C. C. Wong , C. M. Wong , Nat. Commun. 2019, 10, 4681. 31615983 10.1038/s41467-019-12606-7 PMC6794322

[36] N. Kiweler , C. Delbrouck , V. I. Pozdeev , L. Neises , L. Soriano‐Baguet , K. Eiden , F. Xian , M. Benzarti , L. Haase , E. Koncina , M. Schmoetten , C. Jaeger , M. Z. Noman , A. Vazquez , B. Janji , G. Dittmar , D. Brenner , E. Letellier , J. Meiser , Nat. Commun. 2022, 13, 2699. 35577770 10.1038/s41467-022-30363-y PMC9110368

[37] J. Liu , C. Zhang , H. Wu , X. X. Sun , Y. Li , S. Huang , X. Yue , S. E. Lu , Z. Shen , X. Su , E. White , B. G. Haffty , W. Hu , Z. Feng , J. Clin. Invest. 2020, 130, 3253. 32478681 10.1172/JCI132876 PMC7260041

[38] C. Wang , X. Wan , T. Yu , Z. Huang , C. Shen , Q. Qi , S. Xiang , X. Chen , E. Arbely , Z. Q. Ling , C. Y. Liu , W. Yu , Cell Rep. 2020, 32, 108021. 32783943 10.1016/j.celrep.2020.108021

[39] a) R. Dhanasekaran , A. Deutzmann , W. D. Mahauad‐Fernandez , A. S. Hansen , A. M. Gouw , D. W. Felsher , Nat. Rev. Clin. Oncol. 2022, 19, 23; 34508258 10.1038/s41571-021-00549-2 PMC9083341

[40] a) K. Shah , S. Panchal , B. Patel , Pharmacol. Res. 2021, 167, 105532; 33677106 10.1016/j.phrs.2021.105532

[41] H. H. Choi , S. Zou , J. L. Wu , H. Wang , L. Phan , K. Li , P. Zhang , D. Chen , Q. Liu , B. Qin , T. A. T. Nguyen , S. J. Yeung , L. Fang , M. H. Lee , Adv. Sci. 2020, 7, 2000681. 10.1002/advs.202000681 PMC7578864 33101846

[42] K. Li , J. L. Wu , B. Qin , Z. Fan , Q. Tang , W. Lu , H. Zhang , F. Xing , M. Meng , S. Zou , W. Wei , H. Chen , J. Cai , H. Wang , H. Zhang , J. Cai , L. Fang , X. Bian , C. Chen , P. Lan , B. Ghesquiere , L. Fang , M. H. Lee , Cell Res. 2020, 30, 163. 31772275 10.1038/s41422-019-0257-1 PMC7015059

[43] L. Fang , W. Lu , H. H. Choi , S. C. Yeung , J. Y. Tung , C. D. Hsiao , E. Fuentes‐Mattei , D. Menter , C. Chen , L. Wang , J. Wang , M. H. Lee , Cancer Cell 2015, 28, 183. 26267535 10.1016/j.ccell.2015.07.004 PMC4560098

[44] X. Meng , J. Peng , X. Xie , F. Yu , W. Wang , Q. Pan , H. Jin , X. Huang , H. Yu , S. Li , D. Feng , Q. Liu , L. Fang , M. H. Lee , Oncogene 2022, 41, 4231. 35906392 10.1038/s41388-022-02413-8 PMC9439952

