# Supplementary material for: eIF3f Mediates SGOC Pathway Reprogramming by Enhancing Deubiquitinating Activity in Colorectal Cancer
Source: Adv Sci (Weinh). 2023 Aug 6;10(27):2300759. doi: 10.1002/advs.202300759 (PMC10520677; doi:10.1002/advs.202300759)
Supplement: Supplementary file 1 — Supporting Information [file ADVS-10-2300759-s001.pdf]

ADVANCED SCIENCE
Open Access

Supporting Information

for *Adv. Sci.*, DOI 10.1002/advs.202300759

eIF3f Mediates SGOC Pathway Reprogramming by Enhancing Deubiquitinating Activity in Colorectal Cancer

Qihao Pan, Fenghai Yu, Huilin Jin, Peng Zhang, Xiaoling Huang, Jingxuan Peng, Xiaoshan Xie, Xiangli Li, Ning Ma, Yue Wei, Weijie Wen, Jieping Zhang, Boyu Zhang, Hongyan Yu, Yuanxun Xiao, Ran-yi Liu, Qingxin Liu, Xiangqi Meng and Mong-Hong Lee**

Supplementary Materials for

eIF3f Mediates SGOC Pathway Reprogramming by Enhancing Deubiquitinating Activity in Colorectal Cancer

Qihao Pan[1,2,3,4]†, Fenghai Yu[2]†, Huilin Jin[2]†, Peng Zhang[1,2,3], Xiaoling Huang[1,2,3], Jingxuan Peng[2], Xiaoshan Xie[2], Xiangli Li[2], Ning Ma[2], Yue Wei[2], Weijie Wen[2], Jieping Zhang[2], Boyu Zhang[1,2,3], Hongyan Yu[5], Yuanxun Xiao[6], Ran-yi Liu[7], Qingxin Liu[1,2,3], Xiangqi Meng[1,2,3]* and Mong-Hong Lee[1,2,3.8,9]*

Methods

Cell Proliferation and colony formation assays

For cell proliferation assay, CRC cells were seeded at a concentration of 3000 cells per well in 96-well plates. Cell viability was measured using CCK8 reagent (Dojindo Molecular Technologies) according to the manufacturer's instructions of Cell Counting Kit-8 (CCK8) Assay. For tumor cell colony-formation assay, cells were cultured in 6-well plates (500–1000 cells per well) in complete medium for 7–12 days, depending on the sizes of the colonies. The cells were then fixed with methanol for 15 minutes and stained using 0.1% crystal violet for 1 hour.

FACS Analysis for Apoptosis Assay

Apoptosis was conducted as previously described.[1] In brief, cells with indicated treatment were determined by two color analysis using propidium iodide (PI) and FITC-conjugated anti Annexin V (BD Pharmingen, USA) according to the manufacturer's instructions. Cells were harvested and washed three times with PBS then cells were stained with PI and FITC-conjugated anti-Annexin V and analyzed with a FACS flow cytometer.

Metabolite measurements and metabolomics

Metabolite measurements and metabolomics were performed according to the protocol described.[2] Briefly, HCT-116 cells (2×10^7) were seeded in two 100-mm dishes and treated as indicated. Then the metabolites were extracted and measured for untargeted metabolomics at Applied Protein Technology (APT, Shanghai).

Immunofluorescence

First, cells for immunofluorescence were fixed with 4% paraformaldehyde for 15 min at room temperature, followed by 2X washes with PBS. Second, cells were permeabilized with 0.2% Triton X-100 in PBS for 15 min. Third, cells were blocked in PBS with 2% BSA for 1 h at room temperature. After blocking, samples were incubated with indicated primary antibodies listed in Table S3 overnight at 4 °C. Incubation of Alexa Fluorconjugated secondary antibodies (Invitrogen) were carried out for 1 h at room temperature. DAPI was then used for counterstaining the nuclei.

Polysome profiling

Isolation of polysome-bound RNA was performed in triplicate as described by Dai et al with a few modifications.[3] Briefly, for polysome profiling, 5×10^7 viable cells were treated with indicated treatment and collected for further lysis. Then cells were lysed by polysome lysis buffer (50mM MOPS, 15mM MgCl2, 150mM NaCl, 100 mg/ml cycloheximide, 0.5% Triton X-100, 1mg/ml Heparin, 200U/ml RNase inhibitor, 2mM PMSF and 1mM Benzamidine) for 10 min on the ice and centrifuged at 13,000 g for 10 min at 4℃. After that, 1ml of cytoplasmic extract was layered onto 11ml of 10%- 50% Sucrose gradient and then centrifuged at 36,000 rpm at 4℃ for 3h in a SW41 rotor (Beckman Coulter, USA). Separated samples were fractionated at 0.75 ml/min through BR-188 Density Gradient Fractionation System (Brandel, USA) and monitored at absorbance 254 nm. Monosome and polysome fractions were collected for RNA isolation to study the relative distribution of PHGDH, MYC and GAPDH mRNAs. The RNA in each fraction was extracted using RNA-Quick Purification Kit (ESscience).

Evaluation of nascent protein synthesis[4]

Appropriated number of HCT116 or RKO cells were plated into 96-well plates in complete media with/without doxycycline induction of eIF3f KD for 48 h. The Click-iT® Plus OPP Alexa Fluor® 488 Protein Synthesis Assay Kit (Life Technologies, Grand Allen, NY) was used as a non-radioactive method for the detection of protein synthesis utilizing fluorescence microscopy and Incucyte S3 imaging. Cells were further applied to fixation and subsequently following the protocol provided by the manufacturer. The Alexa Fluor® 488 fluorescence intensity of all the cells were automatically quantified by high-content imaging, which corresponds to protein synthesis. As a control, cells were treated with cycloheximide for 1 h before OPP addition. The data are presented as the average protein synthesis in bar graphs and representative images of HCT-116 and RKO cells are depicted in the figure.

Data mining

CRC data sets were downloaded from the publicly available TCGA-COAD databases. GSEA was performed by the JAVA program (http://www.broadinstitute.org/gsea) using MSigDB h.Hallmarks signatures gene set collection to analyze RNA-seq data.

Supplemental Figures

Figure S1

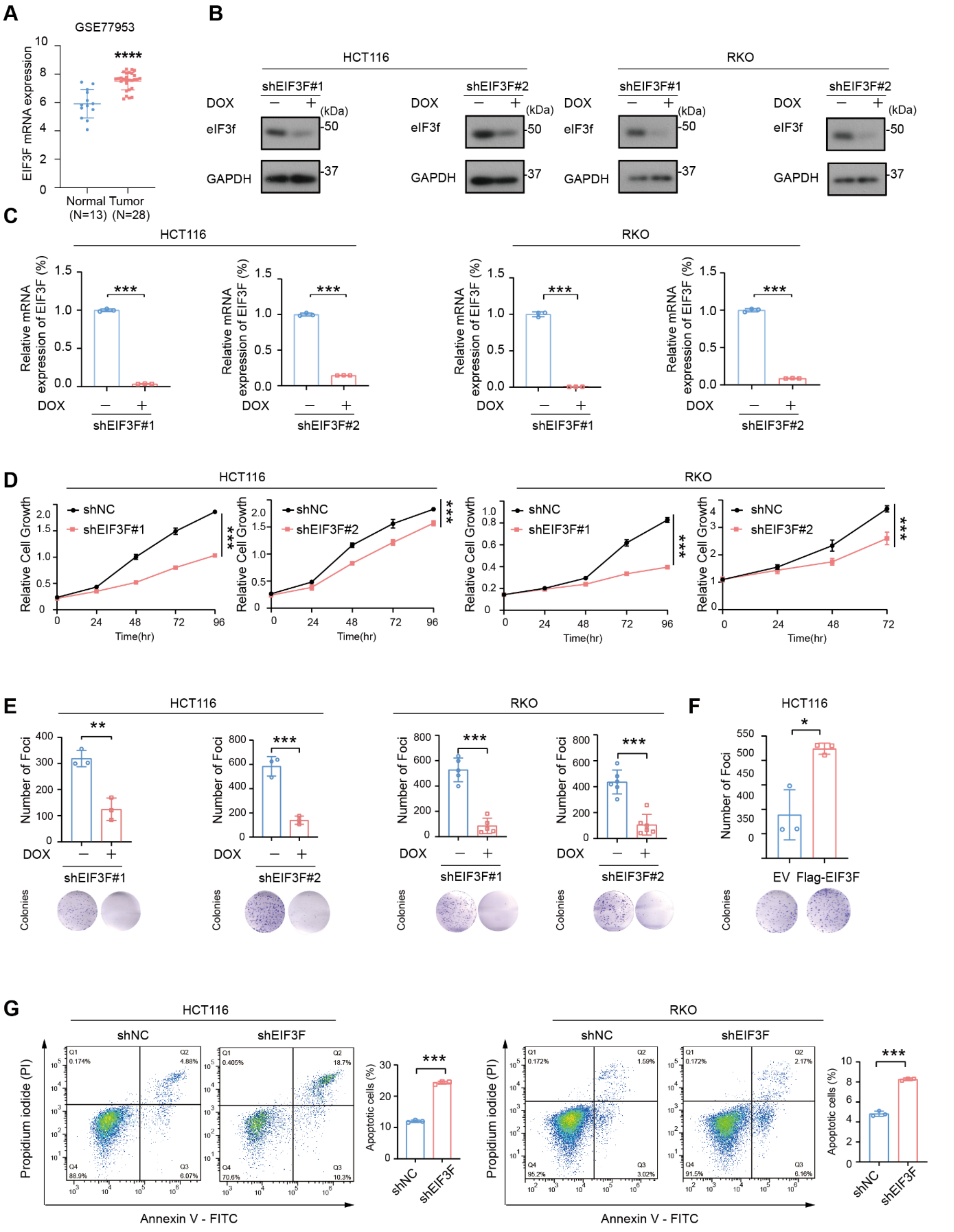

Supplemental Figure S1: eIF3f regulates CRC cell proliferation and apoptosis.
A) Expression level of eIF3f in colorectal tumor and normal tissues (GSE77953). Unpaired student's t test was performed. Solid lines denote the medians, the 5th and 95th percentiles. ****P < 0.0001.
B-C) Immunoblotting and qRT-PCR results showed eIF3f knockdown efficiency of DOX-inducible stable HCT116 cells and RKO cells after treatment with Doxycycline for 72 hours.
D) Growth curves of the cell viability of Dox-inducible pLKO-Tet-On-shEIF3F#1 or pLKO-Tet-On-shEIF3F#2 expressing stable HCT116 cells and RKO cells. Each time point was relative to Time 0 h. The data are presented as the means ± SD. $P<0.05$ represented significant, t-test.
E) Colony formation results of Dox-inducible pLKO-Tet-On-shEIF3F#1 or pLKO-Tet-On-shEIF3F#2 expressing HCT116 cells and RKO cells. The colony number was counted by Image J. The data are presented as the means ± SD. $P<0.05$ was statistically significant, t-test.
F) Colony formation results of pLVX-Flag-EIF3F expressing HCT116 cells. The colony was counted by Image J. The data are presented as the means ± SD. $P<0.05$ was statistically significant, t-test.
G) DOX-induced eIF3f KD leads to apoptosis of indicated cells. Apoptosis was analyzed by annexin V staining.

Figure S2

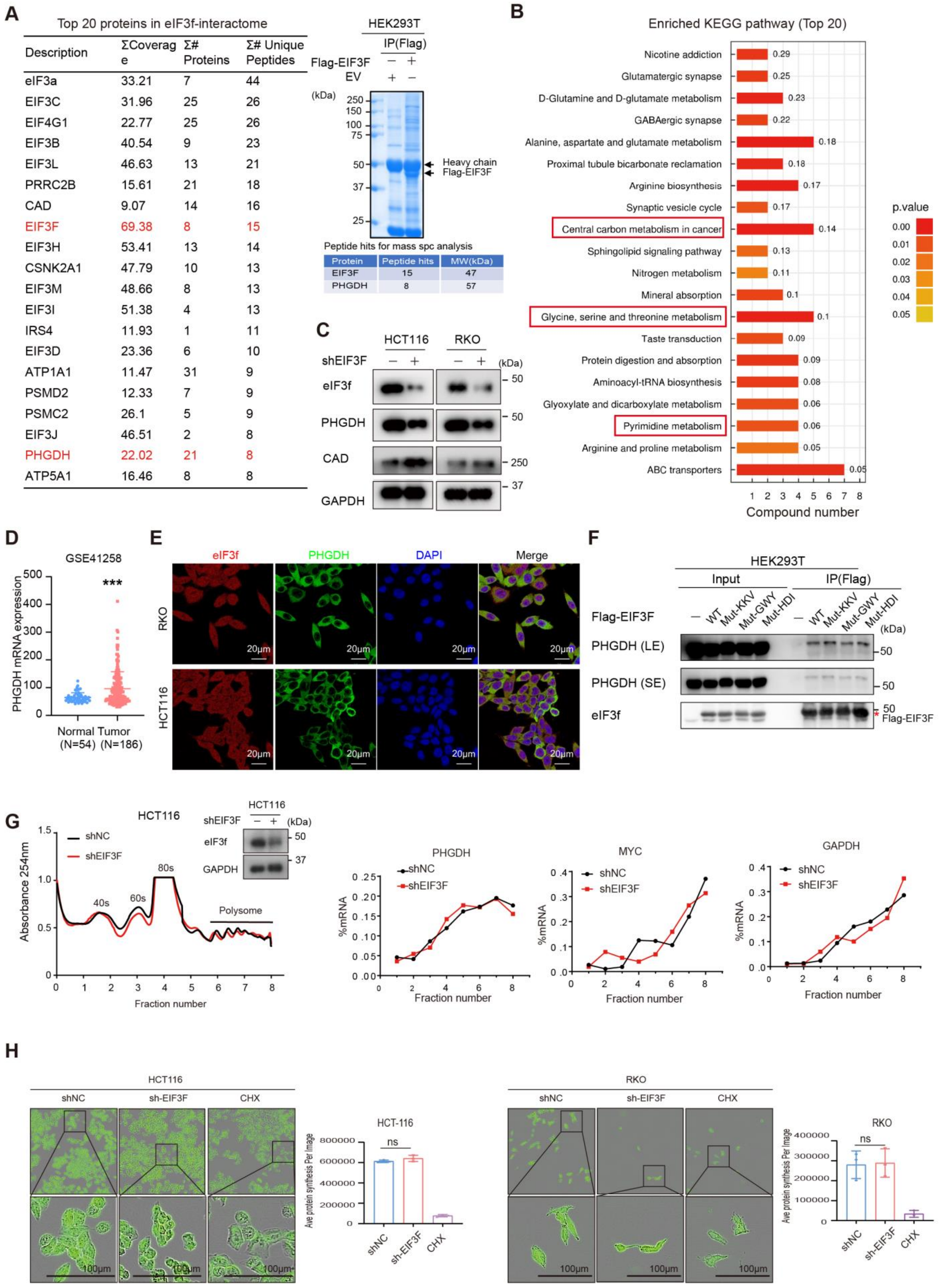

Top 20 proteins in eIF3f-interactome

Description	ΣCoverage	Σ# Proteins	Σ# Unique Peptides
eIF3a	33.21	7	44
EIF3C	31.96	25	26
EIF4G1	22.77	25	26
EIF3B	40.54	9	23
EIF3L	46.63	13	21
PRRC2B	15.61	21	18
CAD	9.07	14	16
EIF3F	69.38	8	15
EIF3H	53.41	13	14
CSNK2A1	47.79	10	13
EIF3M	48.66	8	13
EIF3I	51.38	4	13
IRS4	11.93	1	11
EIF3D	23.36	6	10
ATP1A1	11.47	31	9
PSMD2	12.33	7	9
PSMC2	26.1	5	9
EIF3J	46.51	2	8
PHGDH	22.02	21	8
ATP5A1	16.46	8	8

Peptide hits for mass spc analysis

Protein	Peptide hits	MW(kDa)
EIF3F	15	47
PHGDH	8	57

Supplemental Figure S2:eIF3f is a DUB enzyme for PHGDH.

A) Top 20 interactors in eIF3f-interactome profile. HEK293T cells were transfected either empty vector or Flag-EIF3F 48 hours followed by treatment with 50 μM MG132 for 6 hours before harvesting. Cell lysates were immunoprecipitated with anti-Flag M2 beads and sent for interactome analysis. Coomassie blue staining of the immunoprecipitated profile(Right). The number of LC/MS captured unique peptides of indicated proteins was shown in the table.

B) Untargeted Metabolomics analysis of eIF3f KD cells compared to the control cells was performed. And the top 20 enriched KEGG pathways were shown.

C) Cells were knocked down of eIF3f with doxycycline as previously described and subjected to WB analysis of eIF3f, PHGDH and CAD in HCT-116 and RKO cells.

D) PHGDH mRNA expression was assessed from GSE41258 database. ***P < 0.001.

E) Immunofluorescence colocalization studies of eIF3f and PHGDH in HCT116 and RKO cells. Secondary antibody against primary eIF3f antibody was coupled with Alexa-555, while secondary antibody against primary PHGDH antibody was coupled with Alexa-488. Nuclei were stained with DAPI. Scale bars represent 20μm.

F) Co-immunoprecipitation assay of PHGDH and indicated Flag-EIF3F constructs. '*' indicated exogenous expression of Flag-EIF3F.

G) Polysome profiles of eIF3f KD and control cells. Cells were knocked down of eIF3f as previous described and harvested in indicated time, further lysed and subjected to sucrose gradient centrifugation. The sucrose gradient profiles were obtained by continuous scanning at A254. The positions in the gradients of 40S subunits, 80S ribosomes, and polysomes are labeled (Left). WB analysis of eIF3f KD efficiency was presented as well (Left). The translational status of specific mRNA in eIF3f KD and control cells were examined by qPCR (Right).

H) Evaluation of eIF3f KD on nascent protein synthesis. O-propargyl-puromycin (OPP) was added to the culture medium to label nascent peptides which were visualized by Immunofluorescence microscope after fixation with fluorescent Click iT chemistry in eIF3f KD cells and control cells. The fluorescence intensity of all the cells corresponds to protein synthesis. Representative images of protein synthesis are shown. Data represent means ± SD from three independent experiments.

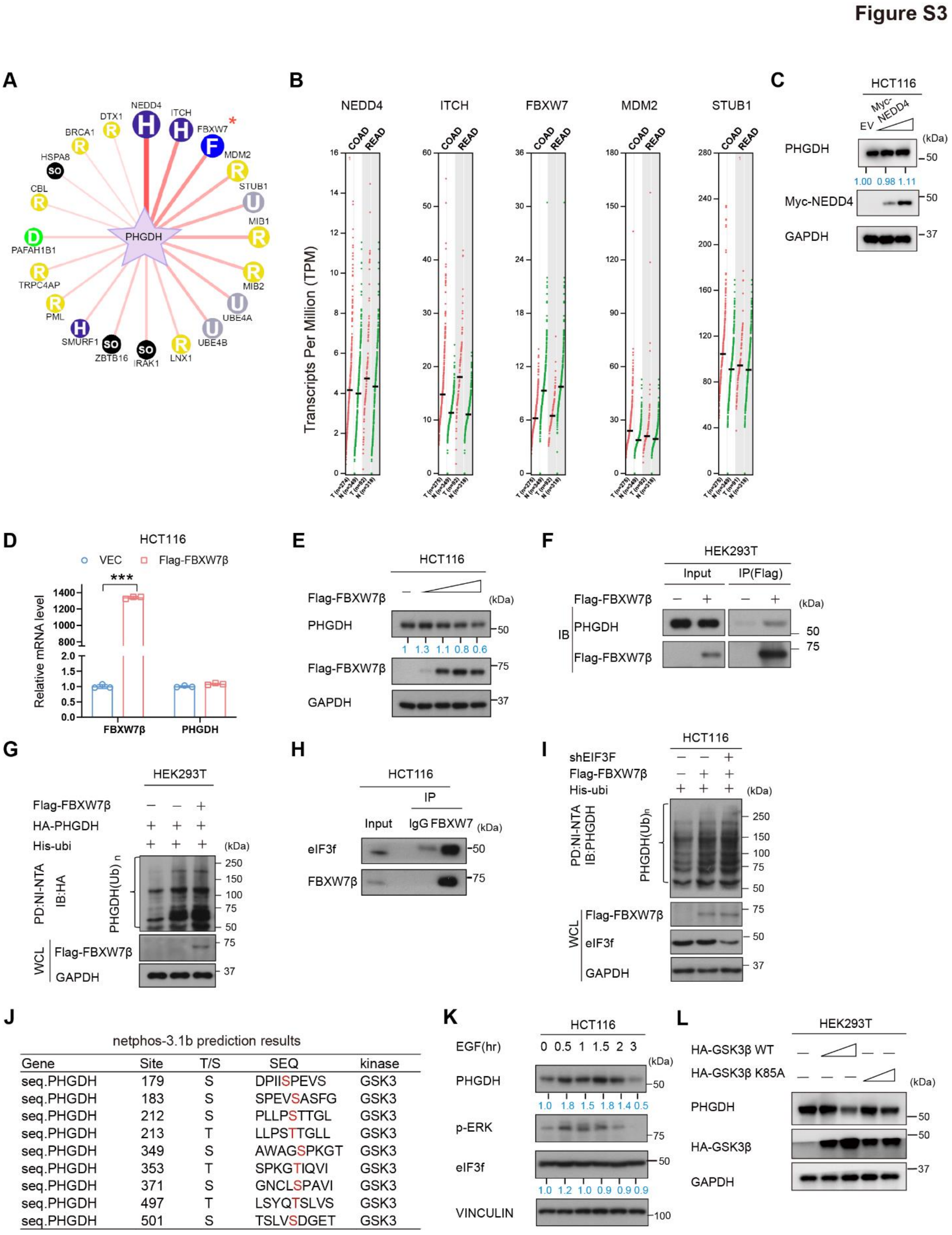

Supplemental Figure S3: PHGDH is a target substrate of FBXW7β.

A) Prediction of potential E3 ligases for PHGDH using Ubibrowser.

B) Potential E3 ligases expressions were assessed from Gepia database.

C) Immunoblotting of PHGDH in HCT-116 cells by transient transfection of NEDD4 overexpression plasmids.

D) FBXW7β doesn't regulate PHGDH mRNA level. HCT116 cells were transfected with empty vector or FBXW7β expression plasmid. 48 hours after transfection, FBXW7 β and PHGDH mRNA level were analyzed via qRT-PCR.

E) FBXW7β decreased steady-state expression of PHGDH protein in HCT-116 cells. Cells were co-transfected with the indicated plasmids. Cell lysates were immunoblotted with indicated antibodies.

F) PHGDH interacted with FBXW7β. Lysates from HEK293T cell expressing empty vector or FBXW7β were immunoprecipitated with M2 beads followed by immunoblotting with PHGDH antibodies.

G) FBXW7 β increases the poly-ubiquitinated level of PHGDH.

H) FBXW7 β interacted with eIF3f endogenously. HCT116 cell lysates were immunoprecipitated with IgG or PHGDH antibody followed by immunoblotting with FBW7 beta antibodies.

I) eIF3f expression level affects FBXW7β-mediated PHGDH polyubiquitination.

J) Netphos analysis(https://services.healthtech.dtu.dk/services/NetPhos-3.1/) of the putative PHGDH phosphorylation sites.

K) EGF treatment leads to change of PHGDH protein expression level.

L) GSK3β increases the steady-state level of PHGDH, while GSK3 β kinase dead mutant (K85A) had marginal effect on PHGDH.

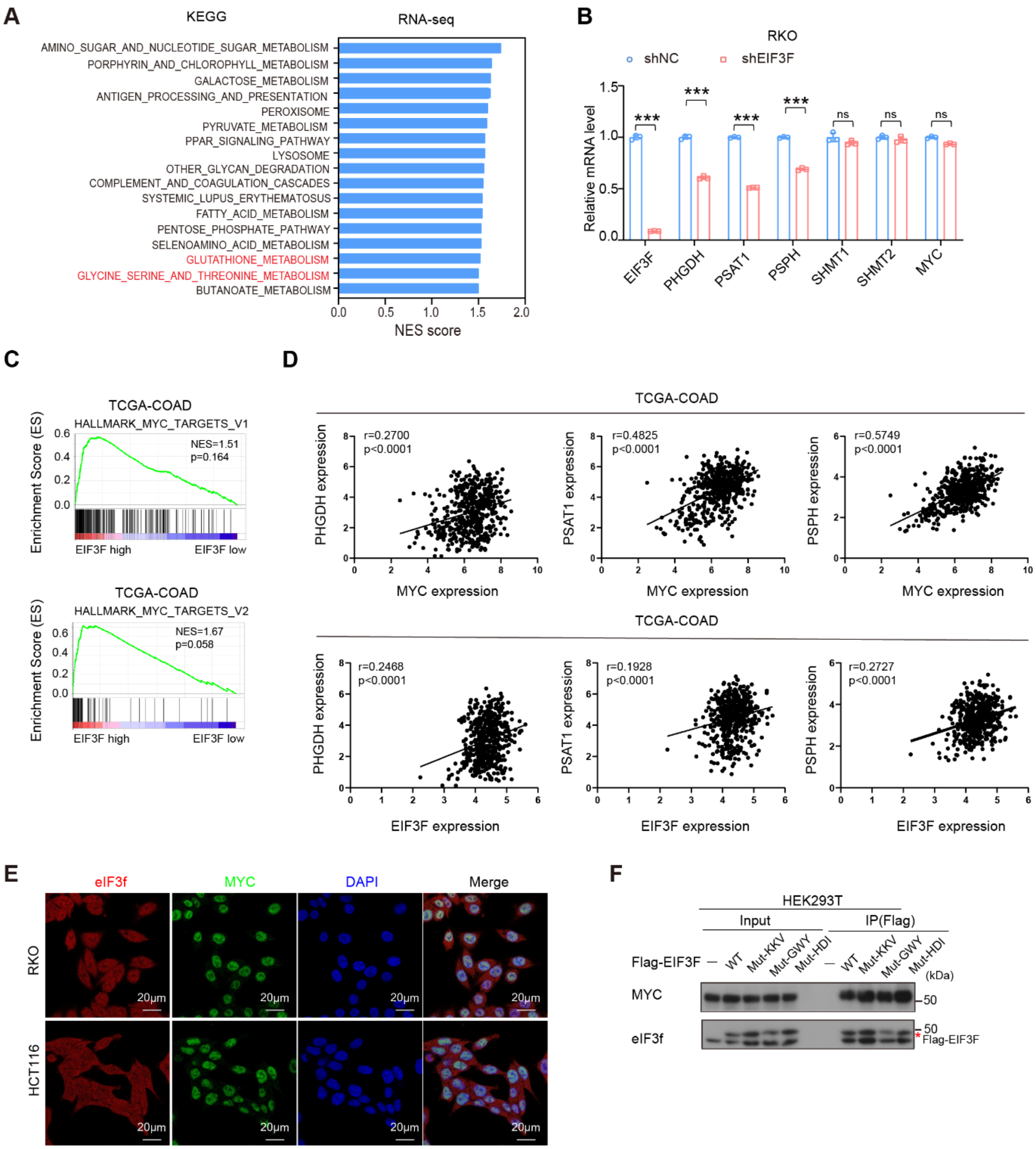

Supplemental Figure S4:eIF3f regulated PHGDH mRNA level through MYC.

A) Glycine-serine and threonine metabolism was significantly changed after **eIF3f** knockdown. The RNA-seq data were analyzed by GSEA program according to KEGG pathway.

B) qRT-PCR results showed that mRNA of the serine synthesis pathway genes and MYC reduced after eIF3f was knocked down in RKO cells.

GSEA analysis of TCGA-COAD data revealed that EIF3F is highly correlated with MYC targeted genes mRNA level.

C-D) Correlation analyses of MYC vs SSP gene expression, or EIF3F vs SSP gene expression based on 521 colon cancer patients' expression data from TCGA.
E) Immunofluorescence colocalization studies of eIF3f and PHGDH in HCT116 and RKO cells. Secondary antibody against primary eIF3f antibody was coupled with Alexa-555, while secondary antibody against primary MYC antibody was coupled with Alexa-488. Nuclei were stained with DAPI. Scale bars represent 20μm.
F) co-immunoprecipitation assay of MYC and indicated Flag-EIF3F constructs. '*' indicated exogenous expression of Flag-EIF3F.

Figure S5

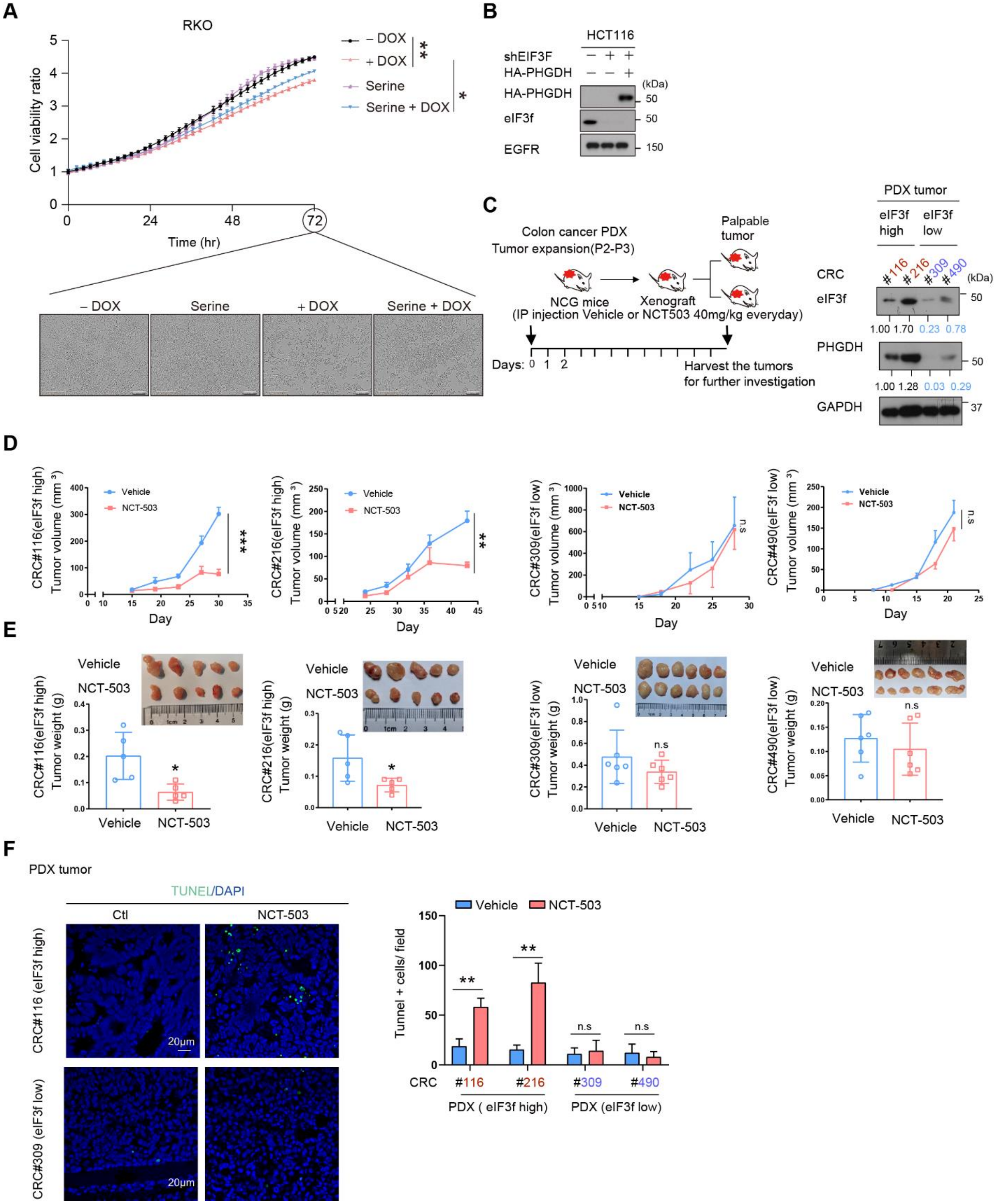

Supplemental Figure S5: NCT-503 inhibits EIF3F high CRC PDX tumors growth.

A) Serine could partially rescue the growth inhibition of CRC cells induced by EIF3F knockdown in RKO cells. IncuCyte was used to measure the confluence of the cells. Each time

point was relative to Time 0 h. TWO WAY-ANOVA test was used to test the significance. * $P < 0.05$.

B) Immunoblotting results showed the knockdown efficiency of eIF3f and overexpression of PHGDH in HCT116 cells used for [U-^{13}C]-labeled glucose tracing experiment.

C) The scheme of patient-derived xenograft (PDX) experiments. The mice were treated with vehicle or NCT-503 (40mg/kg/day) via intraperitoneal injection every day (Left). Immunoblotting of the expression level of EIF3F and PHGDH in PDX tissues (Right).

D-E) Tumor volume and tumor weight of EIF3F high CRC PDX tumor or EIF3F low CRC PDX tumor with NCT503 treatment. Xenograft PDX tumor volume was measured twice a week. **$p < 0.01$, ***$p < 0.001$

F) NCT-503 could induce more apoptotic cells in EIF3F high CRC PDX tumors. Representative immunofluorescent images of apoptotic TUNEL+ signals in CRC PDX tumors. Quantitation of apoptotic TUNEL+ tumor cells in all CRC PDXs after NCT-503 treatment were presented as a bar graph. **$p < 0.01$

SUPPLEMENTARY TABLES

Table S1: List of cloning primers

gene		5'-3'
PCMV5-Flag-EIF3F-WT	Forward	TACAAGGATGACGACGATAAGGATCCCCCGGGATGGCCACACCGGCGGTACCA
	Reverse	GGTTCTAGAATCGATGATATCGAATTCCCCCTATCACAGGTTTACAAGTTTTTC
shEIF3F#1	Forward	CCGGCTCTCAAGTGACTTGCAGCAACTCGAGTTGCTGCAAGTCACTTGAGAGTTTTTG
	Reverse	AATTCAAAAACTCTCAAGTGACTTGCAGCAACTCGAGTTGCTGCAAGTCACTTGAGAG
shEIF3F#2	Forward	CCGGGTACTACGACACTGAACGCATCTCGAGATGCGTTCAGTGTCGTAGTACTTTTTG
	Reverse	AATTCAAAAAGTACTACGACACTGAACGCATCTCGAGATGCGTTCAGTGTCGTAGTAC
PCMV5-Flag-EIF3F-ΔMPN	Forward	GGCGGCCGCGTGGTCAGGCTGATGTTCACGCCTCTGACAGT
	Reverse	ACTGTCAGAGGCGTGAACATCAGCCTGACCACGCGGCCGCC
PCMV5-Flag-EIF3F-ΔC	Forward	TACAAGGATGACGACGATAAGGATCCCCCGGGATGGCCACACCGGCGGTACCA
	Reverse	ACTGTCAGAGGCGTGAACATCAGCCTGACCACGCGGCCGCC
PCMV5-Flag-EIF3F-ΔN	Forward	TACAAGGATGACGACGATAAGGATCCCCCGGGCACCCAGTCATTTTGGCCTC
	Reverse	GGTTCTAGAATCGATGATATCGAATTCCCCCTATCACAGGTTTACAAGTTTTTC
Mut-KKV	Forward	GAACTGCATAGCAGCGCTTCTCCAAATGAGCTCATC
	Reverse	AGAAGCGCTGCTATGCAGTTCATACATATTCTTAGCAAATT
Mut-GWY	Forward	GAGCTCATCCTGGCCTGCTGCGCTACGGGCCATGAC
	Reverse	GCCCGTAGCGCAGCAGGCCAGGATGAGCTCATTTG
Mut-HDI	Forward	ACAGCGGCCTCTGTGCTGGCCGCCGCGTACTACAGCCGAGAGGCCCC
	Reverse	CAGCACAGAGGCCGCTGTGGCGGCAGCGCCCGTAGCGTACCAGCC
PHGDH (179A/183A)	Forward	ATTGCCCCAGAGGTCGCGGCCGCCTTT

		GGTGTTCAGCAGCTGCC
	Reverse	CGCGACCTCTGGGGCAATGATGGGGTCATACCCTATAGTCTTCA
PHGDH (212A/183A)	Forward	GCTCCTCTCCTGCCCGCCACGACAGGCTTGCTGAATGAC
	Reverse	GGCGGGCAGGAGAGGAGCGTGCACAGTGATGAAATCACAGAGA
PHGDH (349A/353A)	Forward	TGCCCCCAAAGGGGCCATCCAGGTGATAACACAGGGAACA
	Reverse	TGGCCCCTTTGGGGGCAGCCCAGGCTCGCATCAG
PHGDH (383A)	Forward	GCAGGCGCCGCAGTCATTGTCGGCCTCCTGAAAGAGGC
	Reverse	AATGACTGCGGCGCCTGCGCAGTTCCCAGCATTCTTCAGG
PHGDH (497A/501A)	Forward	AGGCTTCACTGGTGGCAGATGGGGAGACCTGGCACG
	Reverse	TGCCACCAGTGAAGCCTGGTAGGACAGCAGCCGC
EIF3F-Luc-Reporter	Forward	TCTTACGCGTGCTAGCCCGGGGTGCTAAGAGCTTTACAAGGCAATA
	Reverse	GGGTCAGATCTCGAGCCCGGGACGCCTGTAATCTCAGCACTTCG

Table S2: List of qRT-PCR primers

gene		5'-3'
EIF3F	Forward	CACGCCTCTGACAGTGAAATA
	Reverse	GGGCTAAAGCAGGTCTTCAT
PHGDH	Forward	CACGACAGGCTTGCTGAATGA
	Reverse	CTTCCGTAAACACGTCCAGTG
PSAT1	Forward	ACAGGAGCTTGGTCAGCTAAG
	Reverse	CATGCACCGTCTCATTTGCG
PSPH	Forward	ACGGTGAATATGCAGGTTTTGA
	Reverse	GTTATCCTTGACTTGTTGCCTGA
MYC	Forward	GTCAAGAGGCGAACACACAAC
	Reverse	TTGGACGGACAGGATGTATGC
SHMT1	Forward	TTGCCTCGGAGAATTTCGCC
	Reverse	GTCCCGCCATAGTATCTCTGG
SHMT2	Forward	CCCTTCTGCAACCTCACGAC
	Reverse	TGAGCTTATAGGGCATAGACTCG
β-ACTIN	Forward	TGCTATCCCTGTACGC
	Reverse	TGCCTCAGGGCAGCGGAACC
FBW7	Forward	CCCGAGAAGCGGTTTGATACT
	Reverse	CATGCTCAGGCACGTCAGAA
MYC	Forward	GTCAAGAGGCGAACACACAAC
	Reverse	TTGGACGGACAGGATGTATGC
β-catenin	Forward	CTTCACCTGACAGATCCAAGTC
	Reverse	CCTTCCATCCCTTCCTGTTTAG
TCF4	Forward	GCCTCTTATCACGTACAGCAAT
	Reverse	GCCAGGCGATAGTGGGTAAT
PHGDH-Ebox	Forward	GACCCCTTAGACCGCAGAGGCTG
	Reverse	CGCCAGCAGATACAAAGGCAGAC
pEIF3F-1	Forward	ACAACTCCACTTGAATGTGTAATG
	Reverse	AGCTGTAGGTAGAGCAGGTT

pEIF3F-2	Forward	ACCTGCTCTACCTACAGCTT
	Reverse	AGTCAAGGAGGACTGCAATTT
pEIF3F-3	Forward	TCCTCCTTGACTCCTCTCTTT
	Reverse	TCTTCCCACTTCTCAGAACTTC
pEIF3F-4	Forward	TCTCTCGCCTCCCTCTC
	Reverse	AAGCTCGAGGCTGCAAT

Table S3: List of primary antibodies

antibody	catalog	company	usage
EIF3F（Rabbit）	A303-005A	BETHYL	Western blot (1:2000), IHC (1:2000)
EIF3F（Mouse）	sc-390413	Santa Cruz Biotechnology	Western blot (1:1000), PLA(1:100)
GAPDH（Mouse）	60004-1g	Proteintech Group	Western blot (1:10000)
VINCULIN（Rabbit）	4650s	CST	Western blot (1:2000)
Ki-67 (8D5)	9449	CST	IHC (1:800)
PHGDH（Rabbit）	HPA021241	SIGMA	Western blot (1:2000), IHC (1:1000), PLA(1:100)
PHGDH（Mouse）	sc-100317	Santa Cruz Biotechnology	Western blot (1:1000)
PSAT1（Rabbit）	DF12132	Affinity	Western blot (1:2000)
PSPH（Rabbit）	14513-1-AP	Proteintech Group	Western blot (1:1000)
SHMT2（Rabbit）	DF6347	Affinity	Western blot (1:2000)
FBXW7（Rabbit）	ab109617	Abcam	Western blot (1:2000)
GSK3β（Mouse）	9832S	CST	Western blot (1:2000)
Flag-tag（Mouse）	F1804	SIGMA	Western blot (1:4000)
HA-tag（Rabbit）	51064-2-AP	Proteintech Group	Western blot (1:4000)
Myc-tag（Rabbit）	2276s	CST	Western blot (1:4000)
MYC（Rabbit）	13987S	CST	Western blot (1:2000), PLA(1:100)

[1] H. H. Choi, S. Zou, J. L. Wu, H. Wang, L. Phan, K. Li, P. Zhang, D. Chen, Q. Liu, B. Qin, T. A. T. Nguyen, S. J. Yeung, L. Fang, M. H. Lee, *Adv Sci (Weinh)* **2020**, *7* (20), 2000681, https://doi.org/10.1002/advs.202000681.
[2] W. Yu, Z. Wang, K. Zhang, Z. Chi, T. Xu, D. Jiang, S. Chen, W. Li, X. Yang, X. Zhang, Y. Wu, D. Wang, *Mol Cell* **2019**, *75* (6), 1147, https://doi.org/10.1016/j.molcel.2019.06.039.
[3] Z. Dai, H. Liu, J. Liao, C. Huang, X. Ren, W. Zhu, S. Zhu, B. Peng, S. Li, J. Lai, L. Liang, L. Xu, S. Peng, S. Lin, M. Kuang, *Mol Cell* **2021**, *81* (16), 3339, https://doi.org/10.1016/j.molcel.2021.07.003.
[4] M. K. Altman, A. A. Alshamrani, W. Jia, H. T. Nguyen, J. M. Fambrough, S. K. Tran, M. B. Patel, P. Hoseinzadeh, A. M. Beedle, M. M. Murph, *Cancer Lett* **2015**, *369* (1), 175, https://doi.org/10.1016/j.canlet.2015.08.012.
